# Primary Immunodeficiency Diseases: an Update on the Classification from the International Union of Immunological Societies Expert Committee for Primary Immunodeficiency 2015

**DOI:** 10.1007/s10875-015-0201-1

**Published:** 2015-10-19

**Authors:** Capucine Picard, Waleed Al-Herz, Aziz Bousfiha, Jean-Laurent Casanova, Talal Chatila, Mary Ellen Conley, Charlotte Cunningham-Rundles, Amos Etzioni, Steven M. Holland, Christoph Klein, Shigeaki Nonoyama, Hans D. Ochs, Eric Oksenhendler, Jennifer M. Puck, Kathleen E. Sullivan, Mimi L K. Tang, Jose Luis Franco, H. Bobby Gaspar

**Affiliations:** Laboratory of Human Genetics of Infectious Diseases, Necker Branch, INSERM UMR1163, Necker Hospital for Sick Children, Paris, France; Centre d’étude des déficits immunitaires (CEDI), Hôpital Necker-Enfants Malades, AP-HP, Paris, France; Department of Pediatrics, Faculty of Medicine, Kuwait University, Kuwait City, Kuwait; Allergy and Clinical Immunology Unit, Department of Pediatrics, Al-Sabah Hospital, Kuwait City, Kuwait; Clinical Immunology Unit, Casablanca Children’s Hospital, Ibn Rochd Medical School, King Hassan II University, Casablanca, Morocco; St. Giles Laboratory of Human Genetics of Infectious Diseases, Rockefeller Branch, The Rockefeller University, New York, NY USA; Howard Hughes Medical Institute, New York, NY USA; University Paris Descartes, Imagine Institute, Paris, France; Pediatric Hematology & Immunology Unit, Necker Hospital for Sick Children, Paris, France; Division of Immunology, Children’s Hospital Boston, Boston, MA USA; Department of Medicine and Pediatrics, Mount Sinai School of Medicine, New York, NY USA; Meyer Children’s Hospital-Technion, Haifa, Israel; Laboratory of Clinical Infectious Diseases, National Institute of Allergy and Infectious Diseases, Bethesda, MD USA; Dr von Hauner Children’s Hospital, Ludwig-Maximilians-University Munich, Munich, Germany; Department of Pediatrics, National Defense Medical College, Saitama, Japan; Department of Pediatrics, University of Washington and Seattle Children’s Research Institute, Seattle, WA USA; Department of Clinical Immunology, Hôpital Saint-Louis, Assistance Publique-Hôpitaux de Paris, Paris, France; Université Paris Diderot, Sorbonne Paris Cité, Paris, France; Department of Pediatrics, University of California San Francisco and UCSF Benioff Children’s Hospital, San Francisco, CA USA; Division of Allergy Immunology, Department of Pediatrics, The Children’s Hospital of Philadelphia, Philadelphia, PA USA; Murdoch Childrens Research Institute, Melbourne, VIC Australia; Department of Paediatrics, University of Melbourne, Melbourne, VIC Australia; Department of Allergy and Immunology, Royal Children’s Hospital, Melbourne, Australia; Group of Primary Immunodeficiencies, University of Antioquia, Medellin, Colombia; UCL Institute of Child Health, 30, Guilford Street, London, WC1N 1EH UK

**Keywords:** Primary immunodeficiencies, classification, genetic defects

## Abstract

We report the updated classification of primary immunodeficiencies compiled by the Primary Immunodeficiency Expert Committee (PID EC) of the International Union of Immunological Societies (IUIS). In the two years since the previous version, 34 new gene defects are reported in this updated version. For each disorder, the key clinical and laboratory features are provided. In this new version we continue to see the increasing overlap between immunodeficiency, as manifested by infection and/or malignancy, and immune dysregulation, as manifested by auto-inflammation, auto-immunity, and/or allergy. There is also an increased number of genetic defects that lead to susceptibility to specific organisms which reflects the finely tuned nature of immune defense systems. This classification is the most up to date catalogue of all known and published primary immunodeficiencies and acts as a current reference of the knowledge of these conditions and is an important aid for the genetic and molecular diagnosis of patients with these rare diseases.

## Background

The International Union of Immunological Societies (IUIS) Expert Committee on Primary Immunodeficiency met in London on the 14th and 15th March 2015 to update the classification of human primary immunodeficiencies (PIDs). This report represents the most current and complete catalogue of known PIDs. It serves as a reference for these conditions and provides a framework to help in the diagnostic approach to patients suspected to have PID.

As in previous reports, we have classified the conditions into major groups of PIDs and these are now represented in 9 different tables (Tables 1, 2, 3, 4, 5, 6, 7,8 and 9). In each table, we list the condition, its genetic defect if known and the major immunological and in some conditions the non-immunological abnormalities associated with the disease. This year we have added the gene OMIM number as well as the phenotype OMIM number for ease of reference.

**Table 1:** Immunodeficiencies affecting cellular and humoral immunity

Disease	Genetic defect/Presumed pathogenesis Gene OMIM	Inheritance	Circulating T cells	Circulating B cells	Serum Ig	Associated Features	Phenotype OMIM number
T^−^B^+^ Severe Combined Immunodeficiency (SCID)							
γc deficiency	Mutation of *IL2RG* Defect in γ chain of receptors for IL-2, -4, -7, -9, -15, -21 308380	XL	Markedly decreased	Normal or increased	Decreased	Markedly decreased NK cells;	300400
JAK3 deficiency	Mutation of *JAK3* Defect in Janus activating kinase 3 600173	AR	Markedly decreased	Normal or increased	Decreased	Markedly decreased NK cells;	600802
IL7Rα deficiency	Mutation of *IL7RA* Defect in IL-7 receptor α chain 146661	AR	Markedly decreased	Normal or increased	Decreased	Normal NK cells	608971
CD45 deficiency	Mutation of *PTPRC* Defect in CD45 151460	AR	Markedly decreased	Normal	Decreased	Normal γ/δ T cells	608971
CD3δ deficiency	Mutation of *CD3D* Defect in CD3δ, chain of T cell antigen receptor complex 186790,	AR	Markedly decreased	Normal	Decreased	Normal NK cells No γ/δ T cells	615617
CD3ε deficiency	Mutation of *CD3E* Defect in CD3ε chain of T cell antigen receptor complex 186830,	AR	Markedly decreased	Normal	Decreased	Normal NK cells No γ/δ T cells	615615
CD3ζ deficiency	Mutation of *CD3Z* Defect in CD3ζ chain of T cell antigen receptor complex 186780	AR	Markedly decreased	Normal	Decreased	Normal NK cells No γ/δ T cells	610163
Coronin-1A deficiency	Mutation of *CORO1A* Defective thymic egress of T cells and defective T cell locomotion 605000	AR	Markedly decreased	Normal	Decreased	Detectable thymus EBV-associated B-cell lymphoproliferation	615401
T^−^B^−^ SCID							
DNA recombination defects (for additional DNA repair defects see Table 2)							
RAG 1 deficiency	Mutation of *RAG1* Defective VDJ recombination; defect of recombinase activating gene (RAG) 1 179615	AR	Markedly decreased	Markedly decreased	Decreased		601457
RAG 2 deficiency	Mutation of *RAG2* Defective VDJ recombination; defect of recombinase activating gene (RAG) 2 179616	AR	Markedly decreased	Markedly decreased	Decreased		601457
DCLRE1C (Artemis) deficiency	Mutation of *ARTEMIS* Defective VDJ recombination; defect in Artemis DNA recombinase-repair protein 605988	AR	Markedly decreased	Markedly decreased	Decreased	Radiation sensitivity	602450
DNA PKcs deficiency	Mutation of *PRKDC* Defective VDJ recombination; defect in DNA PKcs Recombinase repair protein 600899	AR	Markedly decreased	Markedly decreased	variable	Radiation sensitivity, microcephaly and developmental defects Autoimmunity and granuloma	615966
Cernunnos/XLF deficiency	Mutation of *Cernunnos* Defective VDJ recombination; defect in Cernunnos 611290	AR	Markedly decreased	Markedly decreased	Decreased	Radiation sensitivity, microcephaly and developmental defects	611291
DNA ligase IV deficiency	Mutation of *LIG4* Defective VDJ recombination; defect in DNA ligase IV 601837	AR	Markedly decreased	Markedly decreased	Decreased	Radiation sensitivity, microcephaly and developmental defects	606593
Reticular dysgenesis, AK2 deficiency	Mutation of *AK2* Defective maturation of lymphoid and myeloid cells (stem cell defect) Defect in mitochondrial adenylate kinase 2. 103020	AR	Markedly decreased	Decreased or normal	Decreased	Granulocytopenia and deafness	267500
Adenosine deaminase (ADA) deficiency	Mutation of *ADA* Absent ADA activity, elevated lymphotoxic metabolites (dATP, S-adenosyl homocysteine) 608958	AR	Absent from birth (null mutations) or progressive decrease	Absent from birth of progressive decrease	Progressive decrease	Decreased NK cells, often with costochondral junction flaring, neurological features, hearing impairment, lung and liver manifestations; partial ADA deficiency may lead to delayed or milder presentation	102700
Combined immunodeficiencies generally less profound than severe combined immunodeficiency							
DOCK2 deficiency	Mutations in *DOCK2* required for RAC1 activation, actin polymerization, T-cell proliferation, chemokine-induced lymphocyte migration and NK-cell degranulation 603122	AR	Decreased. Poor response to PHA. Low TRECs	Normal	Decreased/ Normal. Poor antibody responses	Normal NK numbers, but defective function. Impaired interferon responses in hematopoietic and non-hematopoietic cells	616433
CD40 ligand deficiency	Mutation of *CD40LG* Defects in CD40 ligand (CD40L; also called TNFSF5 or CD154) cause defective isotype switching and impaired dendritic cell signaling 300386	XL	Normal; may progressively decrease	sIgM^+^ and sIgD^+^ B cells present, other surface isotype positive B cells absent	IgM increased or normal, other isotypes decreased	Neutropenia, thrombocytopenia; hemolytic anemia, biliary tract and liver disease, opportunistic infections	308230
CD40 deficiency	Mutation of *CD40 (*also called TNFRSF5) Defects in CD40 cause defective isotype switching and impaired dendritic cell signaling 109535	AR	Normal	IgM^+^ and IgD^+^ B cells present, other isotypes absent	IgM increased or normal, other isotypes decreased	Neutropenia, gastrointestinal and liver/biliary tract disease, opportunistic infections	606843
ICOS deficiency	Mutations in *ICOS;* a co-stimulatory molecule expressed on T cells 604558	AR	Normal	Normal	Low	Recurrent infections; autoimmunity, gastroenteritis, may have granulomas	607594
CD3γ deficiency	Mutation of *CD3G.* Defect in CD3γ component of the T cell antigen receptor complex 186740	AR	Normal, but reduced TCR expression	Normal	Normal		615607
CD8 deficiency	Mutation of *CD8A.* Defects of CD8 α chain, important for maturation and function of CD8 T cells 186910	AR	Absent CD8, normal CD4 cells	Normal	Normal		
ZAP-70 deficiency	Mutation in ZAP70 intracellular signaling kinase, acts downstream of TCR 176947	AR	Decreased CD8, normal CD4 cells	Normal	Normal	Autoimmunity in some cases	269840
MHC class I deficiency	Mutations in *TAP1*, gene, causing MHC class I non-expression 170260	AR	Decreased CD8, normal CD4 cells; absent MHC I expression on lymphocytes	Normal	Normal	Vasculitis; pyoderma gangrenosum	604571
MHC class I deficiency	Mutations in *TAP2*, gene, causing MHC class I non-expression 170261	AR	Decreased CD8, normal CD4 cells; absent MHC I expression on lymphocytes	Normal	Normal	Vasculitis; pyoderma gangrenosum	604571
MHC class I deficiency	Mutations in *TAPBP* (tapasin) gene, causing MHC class I non-expression 601962	AR	Decreased CD8, normal CD4 cells; absent MHC I expression on lymphocytes	Normal	Normal	Vasculitis; pyoderma gangrenosum	604571
MHC class I deficiency	Mutations in *B2M* gene, causing MHC class I non-expression 109700	AR	Decreased CD8, normal CD4 cells; absent MHC I expression on lymphocytes	Normal	Normal	Sinopulmonary infections, cutaneous granuloma, hypoproteinemia. Absent expression of β2m associated proteins like MHC-I, CD1a, and CD1b, CD1c on β2m-deficient cells	not yet assigned
MHC class II deficiency group A	Mutation in transcription factors for MHC class II proteins (*CIITA* gene) 600005	AR	Decreased CD4 cells Absent MHC II expression on lymphocytes	Normal	Normal or decreased	Failure to thrive, diarrhea, respiratory tract infections liver/biliary tract disease	209920
MHC class II deficiency group B	Mutation in transcription factors for MHC class II proteins *RFXANK* gene 603200	AR	Decreased CD4 cells Absent MHC II expression on lymphocytes	Normal	Normal or decreased	Failure to thrive, diarrhea, respiratory tract infections liver/biliary tract disease	209920
MHC class II deficiency group C	Mutation in transcription factors for MHC class II proteins *RFX5*, gene) 601863	AR	Decreased CD4 cells Absent MHC II expression on lymphocytes	Normal	Normal or decreased	Failure to thrive, diarrhea, respiratory tract infections liver/biliary tract disease	209920
MHC class II deficiency group D	Mutation in transcription factors for MHC class II proteins (*RFXAP* gene 601861	AR	Decreased CD4 cells Absent MHC II expression on lymphocytes	Normal	Normal or decreased	Failure to thrive, diarrhea, respiratory tract infections liver/biliary tract disease	209920
ITK deficiency	Mutations in *ITK* encoding IL-2 inducible T cell kinase required for TCR-mediated activation 186973	AR	Progressive decrease	Normal	Normal or decreased	EBV associated B cell lymphop-roliferation, lymphoma Normal or decreased IgG	613011
MAGT1 deficiency	Mutations in *MAGT1*, Impaired Mg^++^ flux leading to impaired TCR signaling 300715	XL	Decreased CD4 cells reduced numbers of RTE, impaired T-cell proliferation in response to CD3	Normal	Normal	EBV infection, lymphoma; viral infections, respiratory and GI infections,	300853
DOCK8 deficiency	Mutations in *DOCK8* encoding a dedicator of cytokinesis regulator of intracellular actin reorganisation 611432	AR	Decreased; Impaired T lymphocyte proliferation; Treg deficiency and poor function	Decreased; low CD27+ memory B cells	Low IgM, increased IgE	Decreased NK cells with impaired function, hypereosinophilia, recurrent infections; severe atopy, extensive cutaneous viral and staphylococcal infections, susceptibility to cancer. Defects in peripheral B tolerance.	243700
RhoH deficiency	Mutations in *RHOH* – an atypical Rho GTPase transducing signals downstream of various membrane receptors 602037	AR	Normal low naïve T cells and RTE, restricted T cell repertoire and impaired T cells proliferation in response to CD3 stimulation.	Normal	Normal	HPV infection, lymphoma, lung granulomas, molluscum contagiosum,	not yet assigned
MST1 deficiency	Mutations in *STK4* – a serine/threonine kinase 604965	AR	Decreased increased proportion of terminal differentiated effector memory cells (TEMRA), low naïve T cells, restricted T cell repertoire in the TEMRA population and impaired T cells proliferation	Decreased	High	Recurrent bacterial, viral, and candidal infections; intermittent neutropenia; EBV-driven lymphoproliferation; lymphoma; Congenital heart disease, autoimmune cytopenias; HPV infection.	614868
TCRα deficiency	Mutations in *TRAC* – essential component of the T cell receptor 186880	AR	Normal All CD3 T cells expressed TCRγδ (or may be better to say: TCRαβ T-cell deficiency), impaired T cells proliferation	Normal	Normal	Recurrent viral, bacterial and fungal infections, immune dysregulation autoimmunity, and diarrhea.	615387
LCK deficiency	Defects in *LCK* – a proximal tyrosine kinase that interacts with TCR 153390	AR	Normal total numbers but CD4+ T-cell lymphopenia, low Treg numbers, restricted T cell repertoire and impaired TCR signaling	Normal	Normal IgG and IgA and increased IgM	Diarrhea, recurrent infections, immune dysregulation autoimmunity,	615758
MALT1 deficiency	Mutations in *MALT1* – a caspase-like cysteine protease that is essential for nuclear factor-kappa-B activation 604860	AR	Normal number but impaired T cells proliferation	Normal	Normal Impaired antibody response	Bacterial, fungal and viral infections	615468
CARD11 deficiency	Defects in *CARD11* – acts as a scaffold for NF-КB activity in the adaptive immune response 607210	AR	Normal predominance of naive T-lymphocyte, impaired T cells proliferation	Normal predominance of transitional B lymphocytes,	Absent/low	Pneumocystis jirovicii pneumonia, bacterial infections,	615206
BCL10 deficiency	Mutations in *BCL10* which encodes the B cell CLL / lymphoma 10 protein that forms a heterotrimer with Malt1 and CARD family adaptors and plays a role in NF-kB signaling 603517	AR	Normal numbers, low memory T and Tregs, decreased proliferation to antigen and anti-CD3	Normal number; decreased memory and switched B cells	Low	Recurrent bacterial and viral infections, candidiasis, gastroenteritis	616098
IL-21 deficiency	Mutation in *IL21* 605384	AR	Normal number. Normal/low function	Low	IgG deficiency	Severe early onset colitis	615767
IL-21R deficiency	Defects in *IL21R* – together with common gamma chain binds IL-21 605383	AR	Abnormal T cell cytokine production; Abnormal T cell proliferation to specific stimuli	Normal	Normal but impaired specific responses	Suspectibility to cryptoporidia and pneumocystis and cholangitis	615207
OX40 deficiency	Defects in *OX40 (TNFRSF4*) encoding a co-stimulatory molecule expressed on activated T cells 600315	AR	Normal T cell numbers; decreased antigen specific memory CD4+ cells	Normal B cell numbers; reduced frequency of memory B cells	Normal	Kaposi’s sarcoma; impaired immunity to HHV8	615593
IKBKB deficiency	Defects in *IKBKB*, encoding IkB 2 kinase 2, a component of the NF-kB pathway 603258	AR	Normal total T cells; absent regulatory and γδ T cells; impaired TCR activation	Normal B cell numbers; impaired BCR activation;	Decreased	Recurrent bacterial, viral and fungal infections; clinical phenotype of SCID	615592
LRBA deficiency	Mutations in *LRBA* (lipopolysaccharide responsive beige-like anchor protein) 606453	AR	Normal or decreased CD4 numbers; T cell dysregulation	Low or normal numbers of B cells	Reduced I IgG and IgA in most	Recurrent infections, inflammatory bowel disease, autoimmunity; EBV infections	614700
CD27 deficiency	Mutations in *CD27* (*TNFRSF7*) encoding TNF-R member superfamily required for generation and long-term maintenance of T cell immunity 186711	AR	Normal	No memory B cells	Hypogamma-globulinaemia following EBV infection	Clinical and immunologic features triggered by EBV infection, HLH Aplastic anaemia, Lymphoma, hypogammaglobulinemia, Low iNKT cells	615122
NIK deficiency	Mutation in *MAP3K14*, encoding NIK (NF-kB-inducing kinase) 604655	AR	Normal number; impaired proliferation in response to antigen stimulation. Polycloncal Vβ repertoires	Decreased total peripheral B cell and switched memory B cells	Hypogamma-globulinaemia	Recurrent bacterial, viral and Cryptosporidium infections. Low NK cell number and defective NK cell activation	Not yet assigned
CTPS1 deficiency	Mutation in *CTPS1*, encoding CTP synthase 1, essential for lymphocyte proliferation 123860	AR	Normal or decreased number Normal or decreased proliferation	Normal/low number	Normal/high IgG	Recurrent/chronic viral infections specially EBV and VZV, bacterial infections, EBV-driven B-cell non-Hodgkin lymphoma	615897
Omenn syndrome	Hypomorphic mutations in *RAG1, RAG2*, *Artemis, IL7RA*, *RMRP, ADA, DNA Ligase IV, IL2RG*, *AK2*, or associated with DiGeorge syndrome; some cases have no defined gene mutation		Present; restricted T cell repertoire and impaired function	Normal or decreased	Decreased, except for increased IgE	Erythroderma, eosinophilia, adenopathies, hepatosplenomegaly	603554

Total no. of genes in Table 1: 49

New genes added: *DOCK2, B2M, IL21*, *MAP3K14, CTPS1*

Notes: Infants with SCID who have maternal T cell engraftment may have allogeneic T cells present even in normal numbers, but that do not function normally; these cells may cause autoimmune cytopenias or graft versus host disease. Hypomorphic mutations in several of the genes that when affected by null mutations cause SCID may result in Omenn syndrome (OS), or “leaky” SCID or a less profound combined immunodeficiency or CID phenotype. Both OS and leaky SCID can be associated with >300 autologous T cells/uL of peripheral blood and reduced rather than absent proliferative responses; Individuals with partially defective, or leaky, mutations are generally more mildly affected compared with those with typical SCID caused by null mutations. A spectrum of clinical findings including typical SCID, OS, leaky SCID, CID, granulomas with T lymphopenia, autoimmunity and CD4+ T lymphopenia can be found in an allelic series of *RAG1* and other SCID associated genes. RAC2 deficiency is a disorder of leukocyte motility and is reported in Table 5; however, one patient with RAC2 deficiency had absent T cell receptor excision circles (TRECs) by newborn screening, though T cell numbers and mitogen responses were not impaired. For additional syndromic conditions with T cell lymphopenia, such as DNA repair defects, cartilage hair hypoplasia, IKAROS deficiency and NEMO syndrome, see Tables 2 and 6; however, it should be noted that individuals with the most severe manifestations of these disorders could have clinical signs and symptoms of SCID

UNC119 deficiency has been removed from this version of the classification tables, as the *UNC119* variant reported previously has been identified as a polymorphism in unaffected individuals (Gorska MM, Alam R. A mutation in the human Uncoordinated 119 gene impairs TCR signaling and is associated with CD4 lymphopenia. *Blood*. 2012 Feb 9;119(6):1399–406. doi: 10.1182/blood-2011-04-350686. Epub 2011 Dec 19). See Erratum (Blood. 2014 Jan 16;123(3):457)

*XL* X-linked inheritance, *AR* autosomal recessive inheritance, *AD* autosomal dominant inheritance, *SCID* severe combined immune deficiency, *EBV* epstein barr virus, *Ca* ^*++*^ calcium, *MHC* major histocompatibility complex, *RTE* recent thymic emigrants, *HPV* human papillomavirus

**Table 2:** Combined immunodeficiencies with associated or syndromic features

Disease	Genetic defect/Presumed pathogenesis OMIM number gene locus	Inheritance	Circulating T cells	Circulating B cells	Serum Ig	Associated features	OMIM number Phenotype
1. Congenital thrombocytopenia							
Wiskott-Aldrich syndrome (WAS)	Mutations in *WAS*; cytoskeletal and immunologic synapse defect affecting haematopoietic stem cell derivatives 301000	XL	Progressive decrease, Abnormal lymphocyte responses to anti-CD3	Normal numbers	Decreased IgM: antibody to polysaccharides particularly decreased; often increased IgA and IgE	Thrombocytopenia with small platelets; eczema; lymphoma; autoimmune disease; IgA nephropathy; bacterial and viral infections. XL thrombocytopenia is a mild form of WAS, and XL neutropenia is caused by missense mutations in the GTPase binding domain of WASP	300392
WIP deficiency	Mutations in *WIPF1*; cytoskeletal and immunologic synapse defect affecting haematopoietic stem cell derivatives 602357	AR	Reduced, Defective lymphocyte responses to anti-CD3	Low	Normal, except for increased IgE	Recurrent infections; eczema; thrombocytopenia. WAS-*like* phenotype.	614493
2. DNA repair defects (other than those in Table 1)							
Ataxia-telangiectasia	Mutations in *ATM*; disorder of cell cycle check-point and DNA double- strand break repair 607585	AR	Progressive decrease, abnormal proliferation to mitogens	Normal	Often decreased IgA, IgE and IgG subclasses; increased IgM monomers; antibodies variably decreased	Ataxia; telangiectasia; pulmonary infections; lymphoreticular and other malignancies; increased alpha fetoprotein and increased radiosensitivity; chromosomal instability	208900
Nijmegen breakage syndrome	Hypomorphic mutations in *NBS1* (*Nibrin*); disorder of cell cycle checkpoint and DNA double- strand break repair 602667	AR	Progressive decrease	Variably reduced	Often decreased IgA, IgE and IgG subclasses; increased IgM; antibodies variably decreased	Microcephaly; bird-like face; lymphomas; solid tumors; increased radiosensitivity; chromosomal instability	251260
Bloom syndrome	Mutations in *BLM (RECQL3*); encoding DNA helicase RecQ protein-like 3 helicase 604610	AR	Normal	Normal	Reduced	Short stature; bird like face; sun-sensitive erythema; marrow failure; leukemia; lymphoma; chromosomal instability	210900
Immunodeficiency with centromeric instability and facial anomalies (ICF1)	Mutations in DNA methyltransferase*DNMT3B* (ICF1) resulting in defective DNA methylation 602900;	AR	Decreased or normal; responses to PHA may be decreased	Decreased or normal	Hypogammaglobulinemia; variable antibody deficiency	Facial dysmorphic features; macroglossia; bacterial/opportunistic infections; malabsorption; cytopenias; malignancies; multiradial configurations of chromosomes 1, 9, 16; no DNA breaks	242860
Immunodeficiency with centromeric instability and facial anomalies (ICF2)	Mutations in *ZBTB24* (ICF2) 614064	AR	Decreased or normal; Responses to PHA may be decreased	Decreased or normal	Hypogammaglobulinemia; variable antibody deficiency	Facial dysmorphic features; macroglossia; bacterial/opportunistic infections; malabsorption; cytopenias; malignancies; multiradial configurations of chromosomes 1, 9, 16;	614069
PMS2 deficiency	Mutations in *PMS2*, resulting in Class Switch recombination deficiency due to impaired mismatch repair 600259	AR	Normal	Reduced B cells, switched and non-switched	Low IgG and IgA, elevated IgM, abnormal antibody responses	Recurrent infections; café-au-lait spots; lymphoma, colorectal carcinoma, brain tumor	276300
RNF168 deficiency	Mutations in *RNF168*, resulting in defective DNA double-strand break repair (RIDDLE syndrome) 612688	AR	Normal	Normal	Low IgG, IgM, or low IgA	Short stature; mild defect of motor control to ataxia; normal intelligence to learning difficulties; mild facial dysmorphism to microcephaly; increased radiosensitivity	611943
MCM4 deficiency	Mutations in *MCM4* (minichromosome maintenance complex component 4) gene involved in DNA replication and repair 602638	AR	Normal	Normal	Normal	Viral infections (EBV, HSV, VZV) Adrenal failure Short stature Low NK cells	609981
3. Thymic defects with additional congenital anomalies							
DiGeorge syndrome*	Contiguous gene deletion in chromosome 22q11.2 or mutation of a gene within this deletion region, *TBX1*, encoding a transcription factor critical for development of thymus and adjacent embryonic structures 602054	*De novo* haplo-insufficiency (majority) or AD; phenocopies may have other as yet undefined genetic lesions	Decreased or normal; 5 % have <1500 CD3 T cells/uL in neonatal period	Normal	Normal or decreased	Hypoparathyroidism, conotruncal cardiac malformation, velopalatal insufficiency, abnormal facies, intellectual disability and other abnormalities; often with 3 Mb interstitial deletion in 22q11.2 (or rarely with intragenic mutation of *TBX1*, deletion in 10p)	188400
CHARGE syndrome due to CHD7 defects	Variable defects of the thymus and associated T cell abnormalities, often due to deletions or mutations in transcription regulator *CHD7*, 608892	*De novo* haplo-insufficiency (majority) or AD	Decreased or normal; response to PHA may be decreased	Normal	Normal or decreased	Coloboma, heart anomaly, choanal atresia, mental retardation, genital and ear anomalies; some are SCID-like and have low TRECs	214800
CHARGE syndrome due to SEMA3E defects	Variable defects of the thymus and associated T cell abnormalities, often due to deletions or mutations in transcription regulator, or semaphorin *SEMA3E* 608166	*De novo* haplo-insufficiency (majority) or AD	Decreased or normal; response to PHA may be decreased	Normal	Normal or decreased	Coloboma, heart anomaly, choanal atresia, mental retardation, genital and ear anomalies; some are SCID-like and have low TRECs	214800
Winged helix deficiency (nude) AAB: syndromic SCID	Defects in forkhead box N1 transcription factor encoded by *FOXN1* 600838	AR	Markedly decreased	Normal	Decreased	Alopecia; nail dystropphy; severe infections abnormal thymic epithelium, impaired T cell maturation	601705
4. Immune-osseous dysplasias							
Cartilage hair hypoplasia	Mutations in *RMRP* (RNase MRP RNA) Involved in processing of mitochondrial RNA and cell cycle control 157660	AR	Varies from severely decreased (SCID) to normal; impaired lymphocyte proliferation	Normal	Normal or reduced antibodies variably decreased	Short-limbed dwarfism with metaphysealdysostosis, sparse hair, bone marrow failure, autoimmunity, susceptibility to lymphoma and other cancers, impaired spermatogenesis, neuronal dysplasia of the intestine	250250
Schimke Immunoosseous Dysplasia	Mutations in *SMARCAL1;* involved in chromatin remodeling 606622	AR	Decreased	Normal	Normal	Short stature, spondiloepiphyseal dysplasia, intrauterine growth retardation, nephropathy; bacterial, viral, fungal infections; may present as SCID; bone marrow failure	242900
5. Hyper-IgE syndromes (HIES)							
AD-HIES (Job or Buckley Syndrome)	Dominant-negative heterozygous mutations in signal transducer and activator of transcription *STAT3* 102582	AD Often *de novo* mutation	Normal overall Th-17 and T-follicular helper cells decreased	Normal; reduced switched and non-switched memory B cells; BAFF expression increased	Elevated IgE; specific antibody production decreased	Distinctive facial features (broad nasal bridge), bacterial infections (boils and pulmonary abscesses, pneumatoceles) due to *S. aureus*, aspergillus, *Pneumocystis jirovecii*; eczema, mucocutaneous candidiasis, hyperextensible joints, osteoporosis and bone fractures, scoliosis, retention of primary teeth, aneurysm formation	147060
Comel-Netherton syndrome	Mutations in *SPINK5* resulting in lack of the serine protease inhibitor LEKTI, expressed in epithelial cells 605010	AR	Normal	Switched and non-switched B cells are reduced	Elevated IgE and IgA Antibody variably decreased	Congenital ichthyosis, bamboo hair, atopic diathesis, increased bacterial infections, failure to thrive	256500
PGM3 deficiency	Mutations inphosphoglycomutase 3 (*PGM3)* associated with a glycosylationand atopy 172100	AR	CD8 and CD4 T cells may be decreased	Reduced B and memory B cells	Normal or elevated Ig’s, elevated IgE; eosinophilia	Severe atopy, autoimmunity, bacterial and viral infections, cognitive impairment, hypomyelination	615816
6. Dyskeratosis congenita (DKC) with bone marrow failure and dysfunctional telomere maintenance							
XL-DKC due to Dyskerin deficiency	Mutations in *DKC1* encoding dyskerin 300126	XL	Progressive decrease	Progressive decrease	Variable hypogammag-lobulinemia	Intrauterine growth retardation, microcephaly, nail dystrophy, recurrent infections, digestive tract involvement, pancytopenia, reduced number and function of NK cells. A severe phenotype with developmental delay and cerebellar hypoplasia is known as Hoyeraal-Hreidarsson Syndrome (HHS)	305000
AR-DKC due to nucleolar protein family A member 2 (NHP2) deficiency	Mutations in *NOLA2* (*NHP2*), component of the H/ACA ribonucleo-protein complex 606470	AR	Decreased	Variable	Variable	Pancytopenia, sparse scalp hair and eyelashes, prominent periorbital telangiectasia, hypoplastic/dysplastic nails	613987
AR-DKC due to nucleolar protein family A member 3 (NHP3) or NOP10 deficiency	Mutation in *NOLA3* (*NOP10, PCFT*), a component of the H/ACA ribonucleo-protein complex 606471	AR	Decreased	Variable	Variable	Pancytopenia, sparse scalp hair and eyelashes, prominent periorbital telangiectasia, hypoplastic/dysplastic nails	224230
AR-DKC due to regulator of telomere elongation (RTEL1) deficiency	Mutation in *RTEL1* encoding regulator of telomere elongation helicase 1 (RTEL1) 608833	AD or AR	Decreased	Variable	Variable	Pancytopenia, sparse scalp hair and eyelashes, prominent periorbital telangiectasia, hypoplastic/dysplastic nails. May present as HHS	615190
AD-DKC due to TERC deficiency	Mutation in *TERC* encoding telomerase RNA component 602322	AD	Variable	Variable	Variable	Reticular hyperpigmentation of the skin, dystrophic nails, osteoporosis premalignant leukokeratosis of the oral mucosa, palmar hyperkeratosis, anemia, pancytopenia. May present as HHS	127550
AD-DKC due to TERT deficiency	Mutation in *TERT* encoding telomerase reverse transcriptase 187270	AD or AR	Variable	Variable	Variable	Reticular hyperpigmentation of the skin, dystrophic nails, osteoporosis premalignant leukokeratosis of the oral mucosa, palmar hyperkeratosis, anemia, pancytopenia. AD version is milder than the AR version which can resemble HHS	613989
AD-DKC due to TINF2 deficiency	Mutation in *TINF2* encoding telomerase interacting factor 2 604319	AD	Variable	Variable	Variable	Reticular hyperpigmentation of the skin, dystrophic nails, osteoporosis premalignant leukokeratosis of the oral mucosa, palmar hyperkeratosis, anemia, pancytopenia. May present as HHS	613990
AD/AR -DKC due to TPP1 deficiency	Mutation in adrenocortical dysplasia homolog (ACD) encoding TPP1 affecting the TELpatch domain resulting in failure to recruit telomerase to telomers 609377	AD/AR	Variable	Variable	Variable	Reticular hyperpigmentation of the skin, dystrophic nails, osteoporosis leukoplakia of the oralmucosa, carcinoma, leukemia palmar hyperkeratosis, anemia, pancytopenia. May present as HHS	
AR-DKC due to DCLRE1B deficiency	Mutation in DCLRE1B/ SNM1/APOLLO: DNA CROSS-LINK REPAIR PROTEIN 1B 609683	AR				dyskeratosis congenita and Hoyeraal-Hreidarsson (HH) syndrome	616353
AR-DKC due to PARN deficiency	Mutation in PARN, POLYADENYLATE-SPECIFIC RIBONUCLEASE 604212	AR					616353
7. Defects of Vitamin B12 and Folate metabolism							
Transcobalamin 2 (TCN2) deficiency	Mutation in *TCN2*; encoding a transporter of cobalamin into blood cells 613441	AR	Normal	Variable	Decreased	Megaloblastic anaemia, pancytopaenia, if untreated for prolonged periods results in mental retardation	275350
SLC46A1/PCFT deficiency causing hereditary folate malabsorbtion	Mutation in *SLC46A1*, encoding a proton coupled folate transporter	AR	Variable numbers and activation profile	Variable	Decreased	Megaloblastic anaemia, failure to thrive, if untreated for prolonged periods results in mental retardation	229050 611672
Methylene-tetrahydrofolate dehydrogenase 1 (MTHFD1) deficiency	Mutations in enzyme encoded by *MTHFD*, essential for processing single-carbon folate derivatives	AR	Low	Low	Decreased	Megaloblastic anaemia, failure to thrive, neutropenia, seizures, mental retardation	601634 172460
8. Anhidrotic ectodermaldysplasia with immunodeficiency (EDA-ID)							
(EDA-ID. NEMO /IKBKG deficiency	Mutations of *NEMO* (*IKBKG*), a modulator of NF-κB activation Defects in *IKBKG*, encoding NEMO, a component of the NF-κB pathway Mutations of NEMO (*IKBKG*), a modulator of NF-κB activation 300248	XL	Normal or decreased; poor CR activation function	Normal Low B memory B cells	Decreased; poor specific antibody responses, absent antibody to polysaccharide antigens	anhidrotic ectodermal dysplasia + specific antibody deficiency (lack of Ab response to polysac-charides) + various infections (mycobacteria and pyogens) Various infections (bacteria, mycobacteria, viruses and fungi); colitis, EDA (not in all patients); conical teeth, variable defects of skin pigmentation, monocyte dysfunction	300291, 300584, 300301 300640
EDA-ID IKBA gain of function mutation	Gain of function mutation in *IKBA (NFKIAB)*, encoding IκBα, a component of the NF-κB pathway Gain-of-function mutation of *IKBA*, resulting in impaired activation of NF-κB 164008	AD	Normal total T cells;; impaired TCR activation	Normal B cell numbers; impaired BCR activation;	Decreased; poor specific antibody responses, absent antibody to polysaccharide antigens	Various infections (bacteria, mycobacteria, viruses and fungi); colitis, EDA (not in all patients); variable defects of skin, hair and teeth, T cell and monocyte dysfunction Anhidrotic ectodermal dysplasia + T cell defect + various infections: Recurrent bacterial, viral and fungal infections;	612132
9. Calcium channel defects							
ORAI-I deficiency	Mutation in *ORAI1*, a Ca^++^ release-activated channel (CRAC) modulatory component 610277	AR	Normal; defective TCR mediated activation	Normal	Normal	Autoimmunity, anhydrotic ectodermic dysplasia, non-progressive myopathy	612782
STIM1 deficiency	Mutations in *STIM1*, a stromal interaction molecule 1 605921	AR	Normal; defective TCR mediated activation	Normal	Normal	Autoimmunity, anhydrotic ectodermal dysplasia, non-progressive myopathy	612783
10. Other defects							
Hepatic veno-occlusive disease with immunodeficiency (VODI)	Mutations in nuclear body protein encoded by *SP110* 604457	AR	Normal (decreased memory T cells)	Normal (decreased memory B cells)	Decreased IgG, IgA, IgM; absent germinal centers and tissue plasma cells	Hepatic veno-occlusive disease; Susceptibility to *Pneumocystis jiroveci* pneumonia, CMV, candida; thrombocytopenia; hepatosplenomegaly; cerebrospinal leukodystropy	235550
Facial dysmorphism, immunodeficiency, livedo, short stature (FILS) syndrome	Mutation in *POLE1*; Defective DNA replication 174762	AR	Low naïve T cells; decreased T cell proliferation	Low memory B cells	Decreased IgM and IgG; Lack of antibodies to polysaccharide antigens	Mild facial dysmorphism (malar hypoplasia, high forehead), livedo, short stature; recurrent upper and lower respiratory tract infections, recurrent pulmonary infections and recurrent meningitis	615139
Immunodeficiency with multiple intestinal atresias	Mutation in *TTC7A* (tetratricopeptide repeat (TPR) domain 7A) protein, of unkown function 609332	AR	Variable, but sometimes absent	Normal	Decreased	Multiple intestinal atresias, often with intrauterine polyhydramnios and early demise; some with SCID phenotype	243150
Vici syndrome due to EPG5 deficiency	Mutations in *EPG5* encoding ectopic P-granules autophagy protein 5, involved in the formation of autolysosomes required for autophagy	AR	Profound depletion of CD4+ cells	Defective	Decreased (particularly IgG2)	Agenesis of the corpus callosum, cataracts, cardiomyopathy, skin hypopigmentation, cleft lip/palate, recurrent infections, chronic mucocutaneous candidiasis	242840 615068
Purine nucleoside phosphorylase (PNP) deficiency	Mutation of *PNP* leading to absent PNP, T cell and neurologic defects from elevated toxic metabolites, especially dGTP 164050	AR	Progressive decrease	Normal	Normal or decreased	Autoimmune haemolytic anemia, neurological impairment	613179
HOIL1 deficiency	Mutation of *HOIL1/RBCK1*, encoding a component of the linear ubiquitination chain assembly complex LUBAC, resulting in impaired activation of NF-κB 610924	AR	Normal numbers,	Normal, but decreased memory B cells	Poor antibody production to polysaccharide antigens	Bacterial infections (pyogens), autoinflammation. amylopectinosis	615895
HOIP deficiency	Mutation of *HOIP1 (/RNF31)*, encoding a component of the linear ubiquitination chain assembly complex LUBAC, resulting in impaired activation of NF-κB 612487	AR	Normal numbers	Normal, but decreased memory B cells	decreased	Bacterial infections (pyogens), autoinflammation. Amylopectinosis, Lymphangiectasia	Not yet assigned
Hennekam-lymphangiectasia-lymphedema syndrome	Mutation of *CCBE1*: (COLLAGEN AND CALCIUM-BINDING EGF DOMAIN-CONTAINING PROTEIN1) 612753	AR	Low/variable	Low/variable	decreased	Lymphangiactasia and lymphedema with facial abnormalities and other dysmorphic features	235510
STAT5b deficiency	Mutations in *STAT5B* signal transducer and transcription factor, essential for normal signaling from IL-2 and 15, key growth factors for T and NK cells, as well as other cytokines 604260	AR	Modestly decreased	Normal	Normal	Growth-hormone insensitive dwarfism, dysmorphic features, eczema, lymphocytic interstitial pneumonitis, autoimmunity	245590

Total no. of genes in Table 2: 45

New genes added: *TPP1, DCLRE1B, PARN, CCBE1, HOIP1, EPG5*

Notes: T and B cell number and function in these disorders exhibit a wide range of abnormality; the most severely affected cases meet diagnostic criteria for SCID or leaky SCID and require immune system restoring therapy such as allogeneic hematopoietic cell transplantation

* Although TBX1 deletions are emphasized, data are lacking that demonstrate that isolated TBX1 haploinsufficiency (affecting solely the gene and none of the surrounding 22q11.2 region) explicitly causes T cell or immunologic deficiency in humans

**Table 3:** Predominantly antibody deficiencies

Disease	Genetic defect/Presumed pathogenesis Gene OMIM	Inheritance	Serum Ig	Associated features	Phenotype OMIM number
1. Severe reduction in all serum immunoglobulin isotypes with profoundly decreased or absent B cells					
BTK deficiency	Mutations in *BTK*, a cytoplasmic tyrosine kinase activated by crosslinking of the BCR 300300	XL	All isotypes decreased in majority of patients; some patients have detectable immunoglobulins	Severe bacterial infections; normal numbers of pro-B cells	300755
μ heavy chain deficiency	Mutations in μ heavy chain (*IGHM*); essential component of the pre-BCR 147020	AR	All isotypes decreased	Severe bacterial infections; normal numbers of pro-B cells	601495
λ5 deficiency	Mutations in λ5 (*IGLL1*); part of the surrogate light chain in the pre-BCR 146770	AR	All isotypes decreased	Severe bacterial infections; normal numbers of pro-B cells	613500
Igα deficiency	Mutations in Igα (*CD79A*); part of the pre-BCR and BCR 112205	AR	All isotypes decreased	Severe bacterial infections; normal numbers of pro-B cells	112205 613501
Igβ deficiency	Mutations in Igb (*CD79B*); part of the pre-BCR and BCR 147245	AR	All isotypes decreased	Severe bacterial infections; normal numbers of pro-B cells	612692
BLNK deficiency	Mutations in *BLNK*; a scaffold protein that binds to BTK 604615	AR	All isotypes decreased	Severe bacterial infections; normal numbers of pro-B cells	613502
PI3KR1 deficiency	Mutations in *PIK3R1;* a kinase involved in signal transduction in multiple cell types. Complete loss of PI3K p85-alpha resulting in complete loss of B cell development 171833	AR	All isotypes decreased	Severe bacterial infections; decreased or absent pro-B cells	615214
E47 transcription factor deficiency	Mutations in *TCF3*; a transcription factor required for control of B cell development 147141	AD	All isotypes decreased	Recurrent bacterial infections	Not yet assigned
Thymoma with immunodeficiency	Unknown	None	One or more isotypes may be decreased	Bacterial and opportunistic infections; autoimmunity; decreased number of pro-B cells	
Disease	Genetic defect/Presumed pathogenesis	Inheritance	Serum Ig	Associated features	OMIM number
2. Severe reduction in at least 2 serum immunoglobulin isotypes with normal or low number of B cells					
Common variable immuno-deficiency disorders	Unknown	Variable	Low IgG and IgA and/or IgM	Clinical phenotypes vary: most have recurrent infections, some have polyclonal lymphoproliferation, autoimmune cytopenias and/or granulomatous disease	
CD19 deficiency	Mutations in *CD19*; transmembrane protein that amplifies signal through BCR 107265	AR	Low IgG and IgA and/or IgM	Recurrent infections; May have glomerulonephritis	613493
CD81 deficiency	Mutations in *CD81*; transmembrane protein that amplifies signal through BCR 186845	AR	Low IgG, low or normal IgA and IgM	Recurrent infections; May have glomerulonephritis	613496
CD20 deficiency	Mutations in *CD20;* a B cell surface receptor involved in B cell development and plasma cell differentiation 112210	AR	Low IgG, normal or elevated IgM and IgA	Recurrent infections	613495
CD21 deficiency	Mutations in *CD21;* also known as complement receptor 2 and forms part of the CD19 complex 120650	AR	Low IgG; impaired anti-pneumococcal response	Recurrent infections	614699
TACI deficiency	Mutations in *TNFRSF13B* (TACI); a TNF receptor family member found on B cells and is a receptor for BAFF and APRIL 604907	AD or AR or complex	Low IgG and IgA and/or IgM	Variable clinical expression	240500
BAFF receptor deficiency	Mutations in *TNFRSF13C* (BAFF-R); a TNF receptor family member found on B cells and is a receptor for BAFF 606269	AR	Low IgG and IgM;	Variable clinical expression	613494
TWEAK deficiency	Mutations in a cytokine *TWEAK (TNFSF12);* TNF-related weak inducer of apoptosis 602695	AD	Low IgM and A; lack of anti-pneumococcal antibody	Pneumonia, bacterial infections, warts; thrombocytopenia. neutropenia	not yet assigned
NFKB2 deficiency	Mutations in *NFKB2*; an essential component of the noncanonical NF-κB pathway	AD	Low IgG and IgA and IgM; very low B cells in some	Recurrent infections; adrenal insufficiency; ACTH deficiency; alopecia	615577
MOGS deficiency	Mutation in mannosyl-oligosaccharide glucosidase 601336	AR	Severe hypogammaglobulinemia;	Bacterial and viral infections; severe neurologic disease; also contains glycosylation type IIb (CDG-IIb),	606056
*TRNT1 deficiency*	Mutation in *TRNT1 a* template-independent RNA polymerase required for the maturation of cytosolic and mitochondrial transfer RNAs (tRNAs) 612907	AR	B cell deficiency and hypogammaglobulinemia	congenital sideroblastic anemia; deafness; developmental delay	616084
TTC37 deficiency	*Mutation in TTC37* gene 614589	AR	Poor antibody response to pneumococcal vaccine	Recurrent bacterial and viral infections; Abnormal hair findings: trichorrhexis nodosa	222470
3. Severe reduction in serum IgG and IgA with normal/elevated IgM and normal numbers of B cells					
AID deficiency	Mutations in *AICDA* gene 605257	AR	IgG and IgA decreased; IgM increased	Bacterial infections; enlarged lymph nodes and germinal centers	605258
UNG deficiency	Mutations in *UNG* 191525	AR	IgG and IgA decreased; IgM increased	Enlarged lymph nodes and germinal centers	608106
INO80	INO80 chromatin remodeling complex; mild DNA repair defect 610169	AR	IgG and IgA decreased; IgM increased	Severe bacterial infections	not yet assigned
MSH6	MSH6 gene defect part of mismatch repair [MMR] machinery); DNA repair defect 600678	AR	Variable IgG, defects; increased IgM in some; normal B cells, low switched memory B cells; Ig-CSR and SHM defects	Family or personal history of cancer	not yet assigned
4. Isotype or light chain deficiencies with generally normal numbers of B cells					
Activated PI3K-δ	Mutation in *PIK3CD;* *p110 encoding for p110 subunit of PI3K* 602839	AD gain of function	Reduced IgG2 and impaired antibody to pneumococci and hemophilus	Respiratory infections, bronchiectasis; autoimmunity; chronic EBV, CMV infection	615513
PI3KR1 loss of function	Mutation in *PIK3R1* leading to mutations in p85α 171833	AD loss of function of p85α (leading to activation of PI3K-δ – as above)	Absent IgA, low IgG	EBV, CMV viremia; growth retardation	616005
Ig heavy chain mutations and deletions	Mutation or chromosomal deletion at 14q32	AR	One or more IgG and/or IgA subclasses as well as IgE may be absent	May be asymptomatic	
IGKC deficiency	Mutations in Kappa constant gene	AR	All immunoglobulins have lambda light chain	Asymptomatic	147200
Isolated IgG subclass deficiency	Unknown	Variable	Reduction in one or more IgG subclass	Usually asymptomatic; a minority may have poor antibody response to specific antigens and recurrent viral/bacterial infections	
IgA with IgG subclass deficiency	Unknown	Variable	Reduced IgA with decrease in one or more IgG subclass	Recurrent bacterial infections	
Specific antibody deficiency with normal Ig concentrations and normal numbers of B cells	Unknown	Variable	Normal	Reduced ability to produce antibodies to specific antigens	
Transient hypogammaglobulinemia of infancy with normal numbers of B cells	Unknown	Variable	IgG and IgA decreased	Normal ability to produce antibodies to vaccine antigens, usually not associated with significant infections	
CARD 11 gain of function	CARD11; scaffold for NF-kB activity in the adaptive immune response; gain of function	AD	Congenital B cell lymphocytosis. High B cell numbers due to constitutive NF-κB activation	Splenomegaly; lymphadenopathy	607210; 606445

Total no. of gene in Table 3: 28

New genes added: *MOGS, TRNT1, TTC37, IN08, MSH6, PI3KR1 AD*

Notes: Several autosomal recessive disorders that might previously have been called CVID have been added to Table 3. CD81 is normally co-expressed with CD19 on the surface of B cells. As for CD19 mutations, mutations in CD81 result in normal numbers of peripheral blood B cells, low serum IgG and an increased incidence of glomerulonephritis

Common Variable Immunodeficiency Disorders (CVID) include several clinical and laboratory phenotypes that may be caused by distinct genetic and/or environmental factors. Some patients with CVID and no known genetic defect have markedly reduced numbers of B cells as well as hypogammaglobulinemia. Alterations in *TNFRSF13B (TACI)* and *TNFRSF13C (BAFF-R)* sequences may represent disease modifying mutations rather than disease causing mutations. A small minority of patients with XLP (Table 4), WHIM syndrome (Table 6), ICF (Table 2), VOD1 (Table 2), thymoma with immunodeficiency (Good syndrome) or myelodysplasia are first seen by an immunologist because of recurrent infections, hypogammaglobulinemia and normal or reduced numbers of B cells

*XL* X-linked inheritance, *AR* autosomal recessive inheritance, *AD* autosomal dominant inheritance*; BTK* Bruton tyrosine kinase, *BLNK* B cell linker protein

*AID* activation-induced cytidine deaminase, *UNG* uracil-DNA glycosylase, *Ig(κ)* immunoglobulin or κ light-chain type

**Table 4:** Diseases of immune dysregulation

Disease	Genetic defect/Presumed pathogenesis Gene OMIM	Inheritance	Circulating T Cells	Circulating B cells	Functional defect	Associated Features	Phenotype OMIM number
1. Familial hemophagocytic lymphohistiocytosis (FHL) syndromes							
1.1. FHL syndromes without hypopigmentation							
Perforin deficiency (FHL2)	Mutations in *PRF1*; perforin is a major cytolytic protein 170280	AR	Increased activated T cells	Normal	Decreased to absent NK and CTL activities cytotoxicity	Fever, Hepato-Splenomegaly (HSMG), Hemophagocytic lymphohistiocytosis (HLH), Cytopenias	603553
(UNC13D / Munc13-4 deficiency (FHL3)	Mutations in *UNC13D*; required to prime vesicles for fusion 608897	AR	Increased activated T cells	Normal	Decreased to absent NK and CTL activities (cytotoxicity and/or degranulation)	Fever, HSMG, HLH, Cytopenias,	608898
Syntaxin 11 deficiency, (FHL4)	Mutations in *STX11*, required for secretory vesicle fusion with the cell membrane 605014	AR	Increased activated T cells	Normal	Decreased NK activity (cytotoxicity and/or degranulation)	Fever, HSMG, HLH, Cytopenias,	603552
STXBP2 / Munc18-2 deficiency (FHL5)	Mutations in *STXBP2*, required for secretory vesicle fusion with the cell membrane 601717	AR or AD	Increased activated T cells	Normal	Decreased NK and CTL activities (cytotoxicity and/or degranulation)	Fever, HSMG, HLH, Cytopenias,	613101
SH2D1A deficiency (XLP1)	Mutations in *SH2D1A* encoding an adaptor protein regulating intracellular signaling 300490	XL	Normal or increased activated T cells	Reduced Memory B cells	partially defective NK cell and CTL cytotoxic activity	Clinical and immunologic features triggered by EBV infection: HLH, lymphoproliferation, Aplastic anaemia, lymphoma. Hypogammaglobulinemia, absent iNKT cells	308240
XIAP deficiency (XLP2)	Mutations in *XIAP/ BIRC4* encoding an inhibitor of apoptosis 300079	XL	Normal or Increased activated T cells; low/normal iNK T cells	Normal or reduced Memory B cells	Increased T cells susceptibility to apoptosis to CD95 and enhanced activation-induced cell death (AICD)	EBV infection, Splenomegaly, lymphoproliferation HLH, Colitis, IBD, hepatitis Low iNKT cells	300635
1.2. FHL syndromes with hypopigmentation							
Chediak-Higashi syndrome	Mutations in *LYST*, impaired lysosomal trafficking 606897	AR	Increased activated T cells	Normal	Decreased NK and CTL activities (cytotoxicity and/or degranulation)	Partial albinism, recurrent infections, fever, HSMG, HLH Giant lysosomes, neutropenia, cytopenias, bleeding tendency, progressive neurological dysfunction	214500
Griscelli syndrome, type2	Mutations in *RAB27A* encoding a GTPase that promotes docking of secretory vesicles to the cell membrane 603868	AR	Normal	Normal	Decreased NK and CTL activities (cytotoxicity and/or degranulation)	Partial albinism, fever, HSMG, HLH, cytopenias	607624
Hermansky-Pudlak syndrome, type 2	Mutations in *AP3B1* gene, encoding for the β subunit of the AP-3 complex 603401	AR	Normal	Normal	Decreased NK and CTL activities (cytotoxicity and/or degranulation)	Partial albinism, recurrent infections, pulmonary fibrosis Increased bleeding, neutropenia, HLH	608233
Hermansky-Pudlak syndrome, type 9	Mutations in *PLDN*, encoding Pallidin, a component of the biogenesis of lysosome-related organelles complex-1 (BLOC-1) 604310	AR	(Not assessed; leukopenia)	(Not assessed, leukopenia)	Decreased NK cell cytolytic activity	Oculocutaneous albinism, recurrent cutaneous infections, leukopenia, thrombocytopenia	614171
2. T regulatory cells genetic defects							
IPEX, immune dysregulation, polyendocrinopathy, enteropathy X-linked	Mutations in *FOXP3*, encoding a T cell transcription factor 300292	XL	Normal	Normal	Lack of (and/or impaired function of) CD4^+^ CD25^+^ FOXP3^+^ regulatory T cells (Tregs)	Autoimmune enteropathy, early onset diabetes, thyroiditis hemolytic anemia, thrombocytopenia, eczema Elevated IgE, IgA	304790
CD25 deficiency	Mutations in *IL2RA*, encoding IL-2Rα chain, 147730	AR	Normal to decreased	Normal	No CD4 + C25+ cells with impaired function of Tregs cells	Lymphoproliferation, autoimmunity. Impaired T cell proliferation	606367
CTLA4 deficiency (ALPSV)	Mutations in *CTLA4*, encoding Cytotoxic T Lymphocyte antigen 4, a protein that negatively regulate T cell receptor signaling and T cell activation. 123890	AD	Decreased	Decreased	Impaired function of Treg cells.	Autoimmune cytopenias, enteropathy, interstitial lung disease, extra-lymphoid lymphocytic infiltration recurrent infections,	616100
STAT3 GOF mutations	Mutations in *STAT3*, encoding Signal Transducer and activator 3 102582	AD	Decreased	Decreased	Enhanced STAT3 signaling, leading to increased Th17 cell differentiation, lymphoproliferation and autoimmunity. Decreased Treg cell numbers and impaired phenotype	Lymphoproliferation, Solid organ autoimmunity, recurrent infections.	615952
3. Autoimmunity with or without lymphoproliferation							
APECED (APS-1), autoimmune polyendocrinopathy with candidiasis and ectodermal dystrophy	Mutations in *AIRE*, encoding a transcription regulator needed to establish thymic self-tolerance 607358	AR	Normal	Normal	AIRE-1 serves as check-point in the thymus for negative selection of autoreactive T cells and for generation of Tregs	Autoimmunity: hypoparathyroidism hypothyroidism, adrenal insufficiency, diabetes, gonadal dysfunction and other endocrine abnormalities, chronic mucocutaneous candidiasis, dental enamel hypoplasia, alopecia areata Enteropathy, Pernicious anemia,	240300
ITCH deficiency	Mutations in *ITCH*, an E3 ubiquitin ligase catalyzes the transfer of ubiquitin to a signaling proteins in the cell including phospholipase Cγ1 (PLCγ1) 606409	AR	Not assessed	Not assessed	Itch deficiency may cause immune dysregulation by affecting both anergy induction in auto-reactive effector T cells and generation of Tregs	Early-onset chronic lung disease (interstitial pneumonitis) Autoimmune disorder (thyroiditis, type I diabetes, chronic diarrhea/enteropathy, and hepatitis) Failure to thrive, developmental delay, dysmorphic facial features	613385
Tripeptidyl-Peptidase II Deficiency	Mutations in *TPP2*, encoding tripeptidyl-peptidase II, serine exopeptidase involved in extralysosomal peptide degradation 190470	AR	Decreased	Decreased	TPP2 deficiency results in premature immunosenescence and immune dysregulation	Variable lymphoproliferation, severe autoimmune cytopenias, hypergammaglobulinemia, recurrent infections,	Not yet assigned
3. Autoimmune lymphoproliferative syndrome (ALPS)							
ALPS-FAS	Germinal mutations in *TNFRSF6*, encoding CD95/Fas cell surface apoptosis receptor** 134637	AD AR***	Increased CD4^−^CD8^−^TCRαβ double negative (DN) T cells	Normal, low memory B cells	Apoptosis defect FAS mediated	Splenomegaly, adenopathies, Autoimmune cytopenias, increased lymphoma risk. IgG and A normal or increased Elevated FasL and IL-10, vitamin B12	601859
ALPS-FASLG	Mutations in *TNFSF6*, Fas ligand for CD95 apoptosis 134638	AR	Increased DN T cells	Normal	Apoptosis defect FAS mediated	Splenomegaly, adenopathies, autoimmune cytopenias, SLE; Soluble FasL is not elevated	601859
ALPS-Caspase10	Mutations in *CASP10*, intracellular apoptosis pathway 601762	AD	Increased DN T cells	Normal	Defective lymphocyte apoptosis	Adenopathies, splenomegaly, autoimmunity.	603909
ALPS-Caspase 8	Mutations in *CASP8*, intracellular apoptosis and activation pathways 601763	AR	Slightly increased DN T cells	Normal	Defective lymphocyte apoptosis and activation	Adenopathies, splenomegaly, Bacterial and viral infections, Hypogammaglobulinemia	607271
FADD deficiency	Mutations in *FADD* encoding an adaptor molecule interacting with FAS, and promoting apoptosis 602457	AR	Increased DN T cells	Normal	Defective lymphocyte apoptosis	Functional hyposplenism, Bacterial and viral infections, Recurrent episodes of encephalopathy and liver dysfunction.	613759
PRKC delta deficiency	Mutations in *PRKCD*, encoding a member of the protein kinase C family critical for regulation of cell survival, proliferation and apoptosis 176977	AR	Normal	Low memory B cells and Elevation of CD5 B cells	Apoptotic defect in B cells	Recurrent infections; EBV chronic infection Lymphoproliferation SLE-like autoimmunity (Nephrotic and antiphospholipid syndromes) HypoIgG	615559
4. Immune dysregulation with colitis							
IL-10 deficiency	Mutations in *IL10*, encoding IL-10 124092	AR	Normal	Normal	No functional IL-10 secretion	Inflammatory bowel disease (IBD) Folliculitis, Recurrent respiratory diseases, Arthritis,	not assigned
IL-10Rα deficiency	Mutations in *IL10RA*, encoding IL-10R1 146933	AR	Normal	Normal	Leukocytes no response to IL-10	IBD, Folliculitis, Recurrent respiratory diseases, Arthritis, Lymphoma	613148
IL-10Rβ deficiency	Mutations in *IL10RB*, encoding IL-10R2 123889	AR	Normal	Normal	Leukocytes no response to IL-10, IL-22, IL-26, IL-28A, IL-28B, and IL-29	IBD, Folliculitis, Recurrent respiratory diseases, Arthritis, Lymphoma	612567
NFAT5 haploinsufficiency	Hemizygous deletion of *NFAT5* 604708	AD	Normal	Normal	Decreased memory B cells and plasmablasts	IBD, recurrent sinopulmonary infections	Not yet assigned
5. Type 1 Interferonopathies							
TREX1 deficiency, Aicardi-Goutieres syndrome 1 (AGS1)	Mutations in *TREX1*, encoding nuclease involves in clearing cellular nucleic debris 606609	AR AD*****	Not assessed	Not assessed	Intracellular accumulation of abnormal single-stranded (ss) DNA species leading to increased CSF alpha-IFN production	Progressive encephalopathy Intracranial calcifications, Cerebral atrophy, leukodystrophy, HSMG, Thrombocytopenia, Elevated hepatic transaminases Chronic cerebrospinal fluid (CSF) lymphocytosis	225750
RNASEH2B deficiency, AGS2	Mutations in *RNASEH2B*, encoding nuclease subunit involves in clearing cellular nucleic debris 610326	AR	Not assessed	Not assessed	Intracellular accumulation of abnormal ss-DNA species leading to increased CSF alpha-IFN production	Progressive encephalopathy Intracranial calcifications, Cerebral atrophy, leukodystrophy, HSMG, thrombocytopenia, Elevated hepatic transaminases Chronic CSF lymphocytosis	610181
RNASEH2C deficiency, AGS3	Mutations in *RNASEH2C*, encoding nuclease subunit involves in clearing cellular nucleic debris 610330	AR	Not assessed	Not assessed	Intracellular accumulation of abnormal ss-DNA species leading to increased CSF alpha-IFN production	Progressive encephalopathy Intracranial calcifications, Cerebral atrophy, leukodystrophy, HSMG, thrombocytopenia, Elevated hepatic transaminases Chronic CSF lymphocytosis	610329
RNASEH2A deficienc y, AGS4	Mutations in *RNASEH2A*, encoding nuclease subunit involves in clearing cellular nucleic debris 606034	AR	Not assessed	Not assessed	Intracellular accumulation of abnormal ss-DNA species leading to increased CSF alpha-IFN production	Progressive encephalopathy Intracranial calcifications, Cerebral atrophy, leukodystrophy, HSMG, thrombocytopenia, Elevated hepatic transaminases Chronic CSF lymphocytosis	610333
SAMHD1 deficiency, AGS5	Mutations in *SAMHD1*, encoding negative regulator of the immunostimulatory DNA response 606754	AR	Not assessed	Not assessed	Induction of the cell intrinsic antiviral response, apoptosis, and mitochondrial DNA destruction leading to increased CSF alpha-IFN production	Progressive encephalopathy Intracranial calcifications, Cerebral atrophy, leukodystrophy, HSMG, thrombocytopenia, anemia elevated lactates Chronic CSF lymphocytosis, Skin vascularitis, mouth ulcers, arthropathy	612952
ADAR1 deficiency, AGS6	Mutations in *ADAR1*, encoding a RNA-specific adenosine deaminase 146920	AR	Not assessed	Not assessed	Catalyzes the deamination of adenosine to inosine in dsRNA substrates Markedly elevated CSF IFN-alpha	Progressive encephalopathy intracranial calcification, Severe developmental delay, leukodystrophy	615010
Aicardi-Goutieres syndrome 7 (AGS7)	*IFIH1* 606951	AD	Not assessed	Not assessed	IFIH1 gene encodes a cytoplasmic viral RNA receptor that activates type I interferon signaling through the MAVS adaptor molecule	Progressive encephalopathy intracranial calcification, Severe developmental delay, leukodystrophy	615846
Spondyloenchondro-dysplasia with immune dysregulation (SPENCD)	Mutations in *ACP5*, encoding tartrate-resitant acid phosphatase (TRAP) 171640	AR	Not assessed	Not assessed	Upregulation of IFN-alpha and type I IFN-stimulated genes	Recurrent bacterial and viral infections, Intracranial calcification, SLE-like autoimmunity (Sjögren’s syndrome, hypothyroidism, inflammatory myositis, Raynaud’s disease and vitiligo), hemolytic anemia, thrombocytopenia, skeletal dysplasia, short stature	607944
STING--associated vasculopathy, infantile-onset	*TMEM173 encoding for* STIMULATOR OF INTERFERON GENES 612374	AR	Not assessed	Not assessed	STING activates both the NF-kappa-B and IRF3 transcription pathways to induce expression of IFN-alpha and IFN-beta and exert a potent antiviral effect	Severe infantile-onset autoinfammatory vasculopathy,	615934
ADA2 deficiency	Mutations in CECR1; encoding ADA2 607575	AR	Not assessed	Not assessed	ADAs deactivate extracellular adenosine and terminate signaling through adenosine receptors	Polyarteritis nodosa, childhood-onset, early-onset recurrent ischemic stroke and fever	615688

Total no. of genes in Table 4: 37

New genes added: *PLDN, CTLA4, TPP2, NFAT5, IFIH1, TMEM173, CECR1, STAT 3 (GOF)*

*XL* X-linked inheritance, *AR* autosomal recessive inheritance, *AD* autosomal dominant inheritance, *FHL* familial hemophagocytic lymphohistiocytosis, *HLH* Hemophagocytic lymphohistiocytosis, *HSMG* hepato-splenomegaly, *DN* double-negative, *SLE* systemic lupus erythematous, *IBD* inflammatory bowel disease, *CSF* chronic cerebrospinal fluid

** Somatic mutations of *TNFRSF6* cause a similar phenotype (ALPS-sFAS) see Table 9. Germinal mutation and somatic mutations of *TNFRSF6* can be associated in some ALPS-FAS patients

*** AR ALPS-FAS patients have a most severe clinical phenotype

**** Somatic mutations in KRAS or NRAS can give this clinical phenotype associated auto-immune leukoproliferative disease (RALD) and are now include in Table 9 entitled Phenocopies of PID

***** *de novo* dominant TREX1 mutations have been reported

**Table 5:** Congenital defects of phagocyte number, function, or both

Disease	Genetic defect/ Presumed pathogenesis OMIM gene	Inheritance	Affected cells	Affected function	Associated features	Phenotype OMIM number
1) Congenital neutropenias						
Elastase deficiency (SCN1)	Mutation in *ELANE:* misfolded protein response, increased apoptosis 130130	AD	N	Myeloid differentiation	Susceptibility to MDS/leukemia	202700
GFI 1 deficiency (SCN2)	Mutation in *GFI1*: loss of repression of ELANE 600871	AD	N	Myeloid differentiation	B/T lymphopenia	613107
Kostmann Disease (SCN3)	Mutation in *HAX1*: control of apoptosis 605998	AR	N	Myeloid differentiation	Cognitive and neurological defects in patients with defects in both HAX1 isoforms, susceptibility to MDS/leukemia	610738
G6PC3 deficiency (SCN4)	Mutation in *G6PC3*: abolished enzymatic activity of glucose-6-phosphatase, aberrant glycosylation, and enhanced apoptosis of N and F 611045	AR	N + F	Myeloid differentiation, chemotaxis, O_2_^−^ production	Structural heart defects, urogenital abnormalities, inner ear deafness, and venous angiectasias of trunks and limbs	612541
VPS45 deficiency (SCN5)	Mutation in *VPS45 controls vesicular trafficking* 610035	AR	N+F	Myeloid differentiation, migration	Extramedullary hematopoiesis, bone marrow fibrosis, nephromegaly,	615285
Glycogen storage disease type 1b	Mutation in *G6PT1*: Glucose-6-phosphate transporter 1 602671	AR	N + M	Myeloid differentiation, chemotaxis, O_2_^−^ production	Fasting hypoglycemia, lactic acidosis, hyperlipidemia, hepatomegaly	232220
Cyclic neutropenia	Mutation in *ELANE*: misfolded protein response 130130	AD	N	Differentiation	Oscillations of other leukocytes and platelets	162800
X-linked neutropenia/ myelodysplasia	Mutation in *WAS:* Regulator of actin cytoskeleton (loss of autoinhibition) 300392	XL, gain of function	N + M	Mitosis	Monocytopenia	300299
P14/LAMTOR2 deficiency	Mutation in *ROBLD3/LAMTOR2*: Endosomal adaptor protein 14 610389	AR	N+L Mel	Endosome biogenesis	Neutropenia Hypogammaglobulinemia ↓CD8 cytotoxicity Partial albinism Growth failure	610798
Barth Syndrome	Mutation in Tafazzin (*TAZ*) gene: Abnormal lipid structure of mitochondrial membrane, defective carnitine metabolism 300394	XL	N	Myeloid differentiation	Cardiomyopathy, myopathy, growth retardation	302060
Cohen syndrome	Mutation in *COH1* gene: Pg unknown 607817	AR	N	Myeloid differentiation	Retinopathy, developmental delay, facial dysmorphisms	216550
Clericuzio syndrome Poikiloderma with neutropenia	Mutation in *C16ORF57 (USB1)*, affects genomic integrity 613276	AR	N	Myeloid differentiation	Poikiloderma, MDS	604173
JAGN1 deficiency	Mutations in JAGN1, regulates secretory pathway 616012	AR	N	Myeloid differentiation	Some with a bone phenotype	616022
3-Methylglutaconic aciduria	Mutations in CLPB 616254	AR	N	Myeloid differentiation	Microcephaly, hypoglycemia, hypotonia, ataxia, seizures, cataracts, IUGR	Not yet assigned
G-CSF receptor deficiency	Mutations in CSF3R, the growth factor receptor 138971	AR	N	Myeloid differentiation	Poor response to GCSF	162830
Disease	Genetic defect/ Presumed pathogenesis	Inheritance	Affected cells	Affected function	Associated features	OMIM number
2. Defects of Motility						
Leukocyte adhesion deficiency type 1 (LAD1)	Mutation in *ITGB2*: B chain for adhesion proteins CD18/CD11 600065	AR	N + M + L + NK	Adherence, Chemotaxis, Endocytosis, T/NK cytotoxicity	Delayed cord separation, skin ulcers Periodontitis Leukocytosis	116920
Leukocyte adhesion deficiency type 2 (LAD2)	Mutation in *SLC35C1*: GDP-Fucose transporter 605881	AR	N + M	Rolling, chemotaxis	Mild LAD type 1 features plus hh-blood group plus mental and growth retardation	266265
Leukocyte adhesion deficiency type 3 (LAD3)	Mutation in *KINDLIN3*: Rap1-activation of β1-3 integrins 607901	AR	N + M + L + NK	Adherence, chemotaxis	LAD type 1 plus bleeding tendency	612840
Rac 2 deficiency	Mutation in *RAC2*: Regulation of actin cytoskeleton 602049	AD	N	Adherence, chemotaxis O_2_^−^ production	Poor wound healing, leukocytosis	608203
β-actin deficiency	Mutation in *ACTB*: Cytoplasmic Actin 102630	AD	N + M	Motility	Mental retardation, short stature	243310
Localized juvenile periodontitis	Mutation in *FPR1*: Formylated peptide receptor 136537	AR	N	Formylpeptide induced chemotaxis	Periodontitis only	Not assigned
Papillon-Lefèvre Syndrome	Mutation in *CTSC*: Cathepsin C activation of serine proteases 602365	AR	N + M	Chemotaxis	Periodontitis, palmoplantar hyperkeratosis in some patients	245000
Specific granule deficiency	Mutation in *C/EBPE*: myeloid transcription factor 189965	AR	N	Chemotaxis	Neutrophils with bilobed nuclei	245480
Shwachman-Diamond Syndrome	Mutation in *SBDS:* Defective ribosome synthesis607444	AR	N	Chemotaxis	Pancytopenia, exocrine pancreatic insufficiency, chondrodysplasia	260400
3. Defects of Respiratory Burst						
X-linked chronic granulomatous disease (CGD)	Mutation in *CYBB*: Electron transport protein (gp91phox) 300481	XL	N + M	Killing (faulty O_2_^−^ production)	McLeod phenotype in patients with deletions extending into the contiguous Kell locus	306400
Autosomal recessive CGD	Mutation in *CYBA*: Electron transport protein (p22phox) 608508	AR	N + M	Killing (faulty O_2_^−^ production)	Infections, autoinflammatory phenotype	233690
Autosomal recessive CGD	Mutation in *NCF1*: Adapter protein (p47phox) 608512	AR	N + M	Killing (faulty O_2_^−^ production)	Infections, autoinflammatory phenotype	233700
Autosomal recessive CGD	Mutation in *NCF2*: Activating protein (p67phox) 608515	AR	N + M	Killing (faulty O_2_^−^ production)	Infections, autoinflammatory phenotype	233710
Autosomal recessive CGD	Mutation in *NCF4*: Activating protein (p40 phox) 601488	AR	N + M	Killing (faulty O_2_^−^ production)	Infections, autoinflammatory phenotype	613960
4. Other Defects						
GATA2 deficiency (Mono MAC syndrome)	Mutations in *GATA2*: loss of stem cells 137295	AD	Monocytes + peripheral DC; low NK cells	Multi lineage cytopenias	Susceptibility to *Mycobacteria*, papilloma viruses, histoplasmosis, alveolar proteinosis, MDS/AML/CMML	614286 614172
Pulmonary alveolar proteinosis*	Mutation in *CSF2RA* 306250	Biallelic mutations in pseudoautosomal gene	Alveolar macrophages	GM-CSF signaling	Alveolar proteinosis	300770

Total no. of genes in Table 5: 31

New genes added: *JAGN1, CLBP, CSF3R*

**Table 6:** Defects in Intrinsic and Innate Immunity

Disease	Genetic defect/Presumed pathogenesis OMIM gene	Inheritance	Affected Cell	Functional Defect	Associated Features	Phenotype OMIM Number
1. Medelian Susceptibility to mycobacterial disease (MSMD)						
IL-12 and IL-23 receptor β1 chain deficiency	Mutation in *IL12RB1*: IL-12 and IL-23 receptor β1 chain 601604	AR	L + NK	IFN-γ secretion	Susceptibility to *Mycobacteria* and *Salmonella*	614891
IL-12p40 deficiency	Mutation in *IL12B :* subunit p40 of IL12/IL23 161561	AR	M	IFN-γ secretion	Susceptibility to *Mycobacteria* and *Salmonella*	614890
IFN-γ receptor 1 deficiency	Mutation in *IFNGR1*: IFN-γR ligand binding chain 107470	AR	M + L	IFN-γ binding and signaling	Susceptibility to *Mycobacteria* and *Salmonella*	209950
IFN-γ receptor 1 deficiency	Mutation in *IFNGR1*: IFN-γR ligand binding chain 107470	AD	M + L	IFN-γ binding and signaling	Susceptibility to *Mycobacteria* and *Salmonella*	615978
IFN-γ receptor 2 deficiency	Mutation in *IFNGR2*: IFN-γR accessory chain 147569	AR	M + L	IFN-γ signaling	Susceptibility to *Mycobacteria* and *Salmonella*	614889
STAT1 deficiency (AD form)	Mutation in *STAT1* (lost of function) 600555	AD	M + L	IFN-γsignaling	Susceptibility to *Mycobacteria, Salmonella*	614892
Macrophage gp91 phox deficiency	Mutation in *CYBB*: Electron transport protein (gp 91 phox) 300481	XL	Mϕ only	Killing (faulty O_2_^−^ production)	Isolated susceptibility to mycobacteria	300645
IRF8-deficiency (AD form)	Mutation in *IRF8*: IL12 production by CD1c^+^ MDC 601565	AD	CD1c + MDC	Differentiation of CD1c + MDC subgroup	Susceptibility to *Mycobacteria*	614893
Tyk2 deficiency	Mutation in *TYK2* 176941	AR	Normal, but Multiple cytokine signaling defect	Normal	Susceptibility to intracellular bacteria (Mycobacteria, Salmonella), fungi and viruses (+/−) Elevated IgE	611521
ISG15 deficiency	Mutation in *ISG15* 147571	AR		IFNγ defect production	Susceptibility to Mycobacteria (BCG) Brain calcification	616126
RORc deficiency	Mutation in *RORC* 602943	AR	L + NK	lack of functional RORγT protein : IFNγ defect production complete absence of IL-17A/F-producing T cells	mycobacteriosis and candidiasis	Not yet assigned
2. Epidermodysplasia verruciformis						
EVER1 deficiency	Mutations of *TMC6* 605828	AR	Keratinocytes and leukocytes	EVER proteins may be involved in the regulation of cellular zinc homeostasis in lymphocytes	HPV (group B1) infections and cancer of the skin (typical EV)	226400
EVER2 deficiency	Mutations of *TMC8* 605829	AR	Keratinocytes and leukocytes	EVER proteins may be involved in the regulation of cellular zinc homeostasis in lymphocytes	HPV (group B1) infections and cancer of the skin (typical EV)	226400
WHIM (Warts, Hypogammaglo-bulinemia, infections, Myelokathexis) syndrome	Gain-of-function mutations of *CXCR4*, the receptor for CXCL12 162643	AD	Granulocytes + Lymphocytes	Increased response of the CXCR4 chemokine receptor to its ligand CXCL12 (SDF-1)	warts/Human Papilloma virus (HPV) infection Neutropenia Reduced B cell number Hypogammaglobulinemia	193670
4. Predisposition to severe viral infection						
STAT1 deficiency	Mutations of *STAT1* 600555	AR	T and NK cells and monocytes	STAT1-dependent IFN-α, and -β response	Severe viral infections Mycobacterial infection	613796
STAT2 deficiency	Mutations of *STAT2* 600556	AR	T and NK cells	STAT2-dependent IFN-α, and -β response	Severe viral infections (disseminated vaccine-strain measles)	Not yet assigned
IRF7 deficiency	Mutation in *IRF7* 605047	AR	Leukocytes and plasmacytoid dendritic cells, Non-hematopoietic cells	IFN-α, and -β production IFN-λ production	Severe influenza disease	Not yet assigned
CD16 deficiency	Mutation in CD16 146740	AR	NK cells	Deficient spontaneous NK cell cytotoxicity	Susceptibility to severe viral infections, inc. HSV, EBV, HPV	615707
5. Herpes simplex encephalitis (HSE)						
TLR3 deficiency	(b) Mutations of *TLR3* 603029	AD AR	Central nervous system (CNS) resident cells and fibroblasts	TLR3-dependent IFN-α, -β, and -λ induction	Herpes simplex virus 1 encephalitis (incomplete clinical penetrance for all etiologies listed here)	613002
UNC93B1 deficiency	(a) Mutations of *UNC93B1* 608204	AR	CNS resident cells and fibroblasts	UNC-93B-dependent IFN-α, -β, and -λ induction	Herpes simplex virus 1 encephalitis	610551
TRAF3 deficiency	(c) Mutations of *TRAF3* 601896	AD	CNS resident cells and fibroblasts	TRAF3-dependent IFN-α, -β, and -λ induction	Herpes simplex virus 1 encephalitis	614849
TRIF deficiency	(c) Mutations of *TRIF, also called TICAM1* 607601	AD AR	CNS resident cells and fibroblasts	TRIF-dependent IFN-α, -β, and -λ induction	Herpes simplex virus 1 encephalitis	614850
TBK1 deficiency	(c) Mutations of *TBK1* 604834	AD	CNS resident cells and fibroblasts	TBK1-dependent IFN-α, -β, and -λ induction	Herpes simplex virus 1 encephalitis	Not yet assigned
6. Predisposition to invasive fungal diseases						
CARD9 deficiency	Mutations of *CARD9* 607212	AR	Mononuclear phagocytes	CARD9 signaling pathway	Invasive candidiasis infection Deep dermatophytoses	212050
7. Chronic mucocutaneous candidiasis (CMC)						
IL-17RA deficiency	(a) Mutations in *IL17RA* 605461	AR	Epithelial cells, fibroblasts, mononuclear phagocytes	IL-17RA signaling pathway	CMC Folliculitis	613953
IL-17RC deficiency	Mutations in IL17RC 610925	AR	Epithelial cells, fibroblasts, mononuclear phagocytes	IL-17RC signaling pathway	CMC	Not yet assigned
IL-17F deficiency	(b) Mutations in *IL17F* 606496	AD	T cells	IL-17 F-containing dimers	CMC Folliculitis	613956
STAT1 gain-of-function	(c) gain-of-function mutations in *STAT1* 600555	AD	T cells, B cells, monocytes	Gain-of-function STAT1 mutations that impair the development of IL-17-producing T cells	CMC Various fungal, bacterial and viral (HSV) infections Auto-immunity (Thyroiditis, diabetes, cytopenia) Enteropathy	614162
ACT1 deficiency	(c) Mutations in *ACT1,* also called *TRAF3IP2* (607043)	AR	T cells, fibroblasts	Fibroblasts fail to respond to IL-17A and IL-17 F, and their T cells to IL-17E	CMC Blepharitis, Folliculitis and macroglossia	615527
8. TLR signaling pathway deficiency						
IRAK-4 deficiency	Mutations of *IRAK4*, a component of TLR- and IL-1R-signaling pathway 606883	AR	Lymphocytes + Granulocytes + Monocytes	TIR-IRAK signaling pathway	Bacterial infections (pyogens)	607676
MyD88 deficiency	Mutations of *MYD88*, a component of the TLR and IL-1R signaling pathway 602170	AR	Lymphocytes + Granulocytes + Monocytes	TIR-MyD88 signaling pathway	Bacterial infections (pyogens)	612260
9. Isolated congenital asplenia (ICA)	Mutations in *RPSA* 150370	AD	Spleen	RPSA encodes ribosomal protein SA, a component of the small subunit of the ribosome	Bacteremia (encapsulated bacteria) No spleen	271400
8. Trypanosomiasis	Mutations in *APOL- I* 603743	AD		APOL-I	Trypanosomiasis	Not yet assigned

Total no. of gene defects in Table 6: 32

New genes added : *RORC, IRF7, IL17RC, APOL-1*

*XL* X-linked inheritance, *AR* autosomal recessive inheritance, *AD* autosomal dominant inheritance, *NF-κB* nuclear factor Kappa B, *TIR* Toll and Interleukin 1 Receptor, *IFN* interferon, *HVP* human papilloma virus, *TLR* Toll-like receptor, *IL* interleukin

**Table 7:** Autoinflammatory disorders

Disease	Genetic defect/ Presumed pathogenesis OMIN gene	Inheritance	Affected cells	Functional defects	Associated Features	Phenotype OMIM number
1. Defects effecting the inflammasome						
Familial Mediterranean Fever	Mutations of *MEFV (lead to gain of pyrin function, resulting in inappropriate IL-1β release)* 608107	AR AD	Mature granulocytes, cytokine-activated monocytes.	Decreased production of pyrin permits ASC-induced IL-1 processing and inflammation following subclinical serosal injury; macrophage apoptosis decreased.	Recurrent fever, serositis and inflammation responsive to colchicine. Predisoposes to vasculitis and inflammatory bowel disease.	249100 134610
Mevalonate kinase deficiency (Hyper IgD syndrome)	Mutations of *MVK (lead to a block in the mevalonate pathway. Interleukin-1beta mediates the inflammatory phenotype)* 251170	AR		affecting cholesterol synthesis; pathogenesis of disease unclear	Periodic fever and leukocytosis with high IgD levels	260920
Muckle-Wells syndrome	Mutations of *NLRP3* (also called *NALP3 CIAS1 or PYPAF1*) (*lead to constitutive activation of the NLRP3 inflammasome)* 606416	*AD*	PMNs Monocytes	Defect in cryopyrin, involved in leukocyte apoptosis and NFkB signaling and IL-1 processing	Urticaria, SNHL, amyloidosis.	191900
Familial cold autoinflammatory syndrome 1	Mutations of NLRP3 (See above) 606416	*AD*	*PMNs, monocytes*	same as above	Non-pruritic urticaria, arthritis, chills, fever and leukocytosis after cold exposure.	120100
Familial cold autoinflammatory syndrome 2	Mutations of NLRP12 609648	*AD*	*PMNs, monocytes*	same as above	Non-pruritic urticaria, arthritis, chills, fever and leukocytosis after cold exposure.	611762
Neonatal onset multisystem inflammatory disease (NOMID) or chronic infantile neurologic cutaneous and articular syndrome (CINCA)	Mutations of NLRP3 CIAS1 (See above) 606416	*AD*	*PMNs, chondrocytes*	same as above	Neonatal onset rash, chronic meningitis, and arthropathy with fever and inflammation*.*	607115
NLRC4-MAS (macrophage activating syndrome) Familial cold autoinflammatory syndrome 4	Mutation in NLRC4 (see functional defect) 606831	*AD*	*PMNs monocytes macrophages*	Gain of function mutation in NLRC4 results in elevated secretion of IL-1β and IL-18 as well as macrophage activation	Severe enterocolitis and macrophage activation syndrome	616050 616115
PLAID (PLCγ2 associated antibody deficiency and immune dysregulation) Familial cold autoinflammatory syndrome 3	Mutation in *PLCG2* ((see functional defect) 600220	AD	B cells, NK, Mast cells	Mutations cause activation of IL-1 pathways	Cold urticaria hypogammaglobulinemia	614468
APLAID (autoinflammation and PLCγ2 associated antibody deficiency and immune dysregulation)	Mutation (c2120C > A) in PLCG2 (see function defect) 600220	AD	B cells, NK, mast cells	The mutation leads to activation of the NLRP3 inflammasome (not provoked by cold temperature)	Blistering skin lesion, pulmonary and bowel disease	614878
2. Non inflammasome-related conditions						
(TNF receptor-associated periodic syndrome (TRAPS)	Mutations of *TNFRSF1A* (resulting in increased TNF inflammatory signaling) 191190	AD	PMNs, monocytes	Mutations of 55-kD TNF receptor leading to intracellular receptor retention or diminished soluble cytokine receptor available to bind TNF	Recurrent fever, serositis, rash, and ocular or joint inflammation	142680
Pyogenic sterile arthritis, pyoderma gangrenosum, acne (PAPA) syndrome	Mutations of *PSTPIP1* (also called *C2BP1)* (affects both pyrin and protein tyrosine phosphatase to regulate innate and adaptive immune responses) 606347	AD	Hematopoietic tissues, upregulated in activated T-cells	Disordered actin reorganization leading to compromised physiologic signaling during inflammatory response	Destructive arthritis, inflammatory skin rash, myositis	604416
Blau syndrome	Mutations of *NOD2* (also called CARD15) (involved in various inflammatory processes) 605956	AD	Monocytes	Mutations in nucleotide binding site of CARD15, possibly disrupting interactions with lipopolysaccharides and NF-κB signaling	Uveitis, granulomatous synovitis, camptodactyly, rash and cranial neuropathies, 30 % develop Crohn’s disease	186580
ADAM17 deletion	Mutation in *ADAM17* (leads to tumor necrosis factor α converting enzyme deficiency) 603639	AR	Leukocytes and epithelial cells	Defective TNFα production	Early onset diarrhea and skin lesions	614328
Chronic recurrent multifocal osteomyelitis and congenital dyserythropoietic anemia (Majeed syndrome)	Mutations of *LPIN2* (increased expression of the proinflammatory genes) 605519	AR	Neutrophils, bone marrow cells	undefined	Chronic recurrent multifocal osteomyelitis, transfusion-dependent anemia, cutaneous inflammatory disorders	609628
DIRA (Deficiency of the Interleukin 1 Receptor Antagonist)	Mutations of *IL1RN* (see functional defect) 147679	AR	PMNs, Monocytes	Mutations in the IL1 receptor antagonist allow unopposed action of Interleukin 1	Neonatal onset of sterile multifocal osteomyelitis, periostitis and pustulosis.	612852
DITRA – Deficiency of IL-36 receptor antagonist	Mutation in *IL36RN* (see functional defect) 605507	AR	Keratinocyte Leukocytes	Mutations in IL-36RN leads to increase IL-8 production	Pustular Psoriasis	614204
SLC29A3 mutation	Mutation in *SLC29A3* 612373	AR	Leukocyte, bone cells	Hyperpigmentation hypertrichosis	Histiocytosis-lymphadenopathy plus syndrome	602782
CAMPS (CARD14 mediated psoriasis)	Mutation in *CARD14* (see functional defect) 607211	AD	Mainly in Keratinocyte	Mutations in CARD14 activate the NF-*k*B pathway and production of IL-8	Psoriasis	602723
Cherubism	Mutation in *SH3BP2* (see functional defect) 602104	AD	Stroma cells, bone cells	Hyperactived macrophage and increase NF-kB	Bone degeneration in jaws	118400
CANDLE (chronic atypical neutrophilic dermatitis with lipodystrophy)	Mutation in *PSMB8*, (see functional defect) 177046	AR	Keratinocyte, B cell adipose cells	Mutations cause increase IL-6 production	Dystrophy, panniculitis	256040
COPA defect	Mutation in COPA (Coatamer protein complex, subunit alpha)	AD	PMNs and tissues specific cells	Mutant COPA leads to defective intracellular transport via the coat protein complex I (COPI)	Autoimmune inflammatory arthritis and interstitial lung disease with Th17 dysregulation and autoantibody production	601924

Total no. of gene defects in Table 7: 17

New genes added: *NLRC4, ADAM17, COPA*

Notes: Autoinflammatory diseases are clinical disorders marked by abnormally increased inflammation, mediated predominantly by the cells and molecules of the innate immune system, with a significant host predisposition. While the genetic defect of one of the most common autoinflammatory conditions, PFAPA, is not known, recent studies suggest that it is associated with activation of IL-1 pathway and response to IL-1beta antagonists

Muckle-Wells syndrome, familial cold autoinflammatory syndrome and neonatal onset multisystem inflammatory disease (NOMID) which is also called chronic infantile neurologic cutaneous and articular syndrome (CINCA) are caused by similar mutations in *CIAS1/NLRP3* mutations. The disease phenotype in any individual appears to depend on modifying effects of other genes and environmental factors

*AR* autosomal recessive inheritance, *AD* autosomal dominant inheritance, *PMN* polymorphonuclear cells, *ASC* apoptosis-associated speck-like protein with a caspase recruitment domain, *CARD* caspase recruitment domain, *CD2BP1* CD2 binding protein-1, *PSTPIP1* Proline/serine/threonine phosphatase-interacting protein 1, *SNHL* sensorineural hearing loss, *CIAS1* cold-induced autoinflammatory syndrome 1

**Table 8:** Complement deficiencies

Disease	Genetic defect; presumed pathogenesis OMIM gene	Inheritance	Laboratory features	Associated Features	Phenotype OMIM number
1) Integral complement cascade component deficiencies					
C1q deficiency	*C1QA,*: Classical complement pathway component 120550	AR	Absent CH50 hemolytic activity, Defective activation of the classical pathway Diminished clearance of apoptotic cells	SLE, infections with encapsulated organisms	613652
C1q deficiency	*C1QB:* Classical complement pathway component 120570	AR	Absent CH50 hemolytic activity, Defective activation of the classical pathway Diminished clearance of apoptotic cells	SLE, infections with encapsulated organisms	613652
C1q deficiency	*C1QC:* Classical complement pathway component 120575	AR	Absent CH50 hemolytic activity, Defective activation of the classical pathway Diminished clearance of apoptotic cells	SLE, infections with encapsulated organisms	613652
C1r deficiency	*C1R*: Classical complement pathway component 613785	AR	Absent CH50 hemolytic activity, Defective activation of the classical pathway	SLE, infections with encapsulated organisms	216950
C1s deficiency	*C1S*: Classical complement pathway component 120580	AR	Absent CH50 hemolytic activity Defective activation of the classical pathway	SLE, infections with encapsulated organisms	613783
C4 deficiency	*C4A,* Classical complement pathway components 120810	AR	Absent CH50 hemolytic activity, Defective activation of the classical pathway Complete deficiency requires biallelic mutations/deletions/conversions of both C4A and C4B	SLE, infections with encapsulated organisms	614380
C4 deficiency	*C4B*: Classical complement pathway components 120820	AR	Absent CH50 hemolytic activity, Defective activation of the classical pathway Complete deficiency requires biallelic mutations/deletions/conversions of both C4A and C4B	SLE, infections with encapsulated organisms	614379
C2 deficiency	*C2*: Classical complement pathway component 217000	AR	Absent CH50 hemolytic activity, Defective activation of the classical pathway	SLE, infections with encapsulated organisms, atherosclerosis	613927
C3 deficiency LOF	*C3*: Central complement component 120700	AR	Absent CH50 and AH50 hemolytic activity Defective opsonization Defective humoral immune response	Infections; glomerulonephritis; Atypical Hemolytic-uremic syndrome with gain-of-function mutations.	613779
C3 GOF	*C3*: Central complement component 120700	Gain-of-function AD	Increased activation of complement	Atypical Hemolytic-uremic syndrome	612925
C5 deficiency	*C5*: Terminal complement component 120900	AR	Absent CH50 and AH50 hemolytic activity Defective bactericidal activity	Neisserial infections	609536
C6 deficiency	*C6*: Terminal complement component 217050	AR	Absent CH50 and AH50 hemolytic activity Defective bactericidal activity	Neisserial infections	612446
C7 deficiency	*C7*: Terminal complement component 217070	AR	Absent CH50 and AH50 hemolytic activity Defective bactericidal activity	Neisserial infections	610102
C8 αdeficiency	*C8A*: Terminal complement component 120950	AR	Absent CH50 and AH50 hemolytic activity Defective bactericidal activity	Neisserial infections	613790
C8γ deficiency	*C8G:* Terminal complement component 120930	AR	Absent CH50 and AH50 hemolytic activity Defective bactericidal activity	Neisserial infections	613790
C8β deficiency	*C8B*: Terminal complement component 120960	AR	Absent CH50 and AH50 hemolytic activity Defective bactericidal activity	Neisserial infections	613789
C9 deficiency	*C9*: Terminal complement component 120940	AR	Reduced CH50 and AP50 hemolytic activity Deficient bactericidal activity	Mild susceptibility to Neisserial infections	613825
MASP2 deficiency	*MASP2*: Cleavage of C4 605102	AR	Deficient activation of the lectin activation pathway	Pyogenic infections; Inflammatory lung disease, autoimmunity	613791
Ficolin 3 deficiency	*FCN3:* Activates the classical complement pathway 604973	AR	Absence of complement activation by the Ficolin 3 pathway.	Respiratory infections, abscesses	613860
2) Complement Regulatory defects					
C1 inhibitor deficiency	*SERPING1*: regulation of kinins and complement activation 606860	AD	Spontaneous activation of the complement pathway with consumption of C4/C2 Spontaneous activation of the contact system with generation of bradykinin from high molecular weight kininogen	Hereditary angioedema	106100
Factor B	*CFB:* Activation of the alternative pathway 138470	AD	Gain-of-function mutation with increased spontaneous AH50	aHUS	612924
Factor D deficiency	*CFD*: Regulation of the alternative complement pathway 134350	AR	Absent AH50 hemolytic activity	Neisserial infections	613912
Properdin deficiency	*CFP*: Regulation of the alternative complement pathway 300383	XL	Absent AH50 hemolytic activity	Neisserial infections	312060
Factor I deficiency	*CFI*: Regulation of the alternative complement pathway 217030	AR	Spontaneous activation of the alternative complement pathway with consumption of C3	Infections, Neisserial infections, aHUS, preeclampsia	610984 612923
Factor H deficiency	*CFH*: Regulation of the alternative complement pathway 134370	AR/AD	Spontaneous activation of the alternative complement pathway with consumption of C3	Infections, Neisserial infections, aHUS, preeclampsia	609814 235400
Factor H –related protein deficiencies	*CFHR1-5*: Bind C3b 134371 600889 605336 605337 608593	AR/AD	Normal CH50, AH50, autoantibodies to Factor H. Linked deletions of one or more CFHR genes leads to susceptibility autoantibody-mediated aHUS	aHUS, Neisserial infections	235400
Thrombomodulin	*THBD*: Regulates complement and coagulant activation 188040	AD	Normal CH50, AH50	aHUS	612926
Complement Receptor 3 (CR3) deficiency	*ITGAM* 120980	AR	CR3 expression is lost in LAD1. See LAD1 in Table 5	Infections	609939
Membrane Cofactor Protein (CD46) deficiency	*CD46*: Dissociates C3b and C4b 120920	AD	Inhibitor of complement alternate pathway, decreased C3b binding	aHUS, infections, preeclampsia	612922
Membrane Attack Complex Inhibitor (CD59) deficiency	*CD59:* Regulates the membrane attack complex formation 107271	AR	Erythrocytes highly susceptible to complement-mediated lysis	Hemolytic anemia, polyneuropathy	612300

Total no. of genes Tables 8 and 9: 30

No new genes added to the 2015 classification

*XL* X-linked inheritance, *AR* autosomal recessive inheritance, *AD* autosomal dominant inheritance, *MAC* membrane attack complex, *SLE* systemic lupus erythematosus, *MASP* MBP associated serine protease 2

**Table 9:** Phenocopies of PID

Disease	Genetic defect/presumed pathogenesis	Circulating T cells	Circulating B cells	Serum Ig	Associated features/similar PID
Associated with somatic mutations					
Autoimmune lymphoproliferative syndrome (ALPS–SFAS)	Somatic mutation in *TNFRSF6*	Increased CD4−CD8−double negative (DN) T alpha/beta cells	Normal, but increased number of CD5+ B cells	Normal or increased	Splenomegaly, lymphadenopathy, autoimmune cytopenias Defective lymphocyte apoptosis/*ALPS–FAS (=ALPS type Im)*
RAS-associated autoimmune leukoproliferative disease (RALD)	Somatic mutation in *KRAS* (gain-of-function)	Normal	B cell lymphocytosis	Normal or increased	Splenomegaly, lymphadenopathy, autoimmune cytopenias, granulocytosis, monocytosis/*ALPS-like*
RAS-associated autoimmune leukoproliferative disease (RALD)	Somatic mutation in *NRAS* (gain-of-function)	Increased CD4−CD8−double negative (DN) T alpha/beta cells	Lymphocytosis		Splenomegaly, lymphadenopathy, autoantibodies/*ALPS-like*
Cryopyrinopathy, (Muckle-Wells /CINCA/NOMID-like syndrome)	Somatic mutation in *NLRP3*	Normal	Normal	Normal	Urticaria-like rash, arthropathy, neurological symptoms
Associated with autoantibodies					
Chronic mucocutaneous candidiasis (isolated or with APECED syndrome)	Germline mutation in *AIRE* AutoAb to IL-17 and/or IL-22	Normal	Normal	Normal	Endocrinopathy, chronic mucocutaneous candidiasis/*CMC*
Adult-onset immunodeficiency	AutoAb to IFN gamma	Decreased naive T cells	Normal	Normal	Mycobacterial, fungal, *Salmonella* VZV infections/*MSMD, or CID*
Recurrent skin infection	AutoAb to IL-6	Normal	Normal	Normal	Staphylococcal infections/*STAT3 deficiency*
Pulmonary alveolar proteinosis	AutoAb to GM-CSF	Normal	Normal	Normal	Pulmonary alveolar proteinosis, cryptococcal meningitis/*CSF2RA deficiency*
Acquired angioedema	AutoAb to CI inhibitor	Normal	Normal	Normal	Angioedema/*C1 INH deficiency* (hereditary angioedema)
Atypical Hemolytic Uremic Syndrome	AutoAb to Complement Factor H	Normal	Normal	Normal	aHUS Spontaneous activation of the alternative complement pathway

The classification this year differs in a number of ways from the previous edition published in 2014. Importantly, each defect is now listed in only one table. The diverse immunological phenotypes of many conditions imply that a very large number of conditions could very readily be listed in multiple tables. However, with the increasing number of identified defects, this would make each table large and cumbersome. For this reason, we chose to list each defect in one table only and to place it according to the most pronounced and fundamental defect. For this reason and as an example, CD40L deficiency is now found in Table 1 amongst combined immunodeficiencies, because CD40L is a T cell signaling molecule whose absence leads to both cellular and humoral defects, even though it was originally described as an antibody deficiency. Although some of our placements may be disputed, the committee came to these decisions after much thought and deliberation.

The title of Table 6 has now been slightly changed to ‘Defects in intrinsic and innate immunity’ and contains defects characterized by susceptibility to specific organisms. For this reason, the MSMDs (Mendelian Susceptibility to Mycobacterial Disease) are now in Table 6, having previously been in Table 5 (Phagocytic Disorders).

In previous editions, we have placed an asterisk against conditions in which 10 or fewer individuals had been described in the literature. However, this is now felt to be an artificial indicator as, once described, a condition may be found in additional patients but not necessarily reported. For this reason, there is no specific indicator of the number of patients identified or reported.

There is a growing appreciation of wide phenotypic variability for many of the individual specific gene defects, reflecting not only the variety of mutations within each gene but also host and/or environmental modifying factors that may impact the phenotype even between individuals with the same mutation within the same gene. The complexities of these conditions in terms of clinical and immunological presentation and heterogeneity cannot easily be captured in the limited space of a table format. For this reason, the furthest right column contains the Online Mendelian Inheritance in Man (OMIM) reference for each condition to allow access to a source of greater detail and updated information as to the phenotype.

A number of the new genes included in this edition of the classification tables are molecules associated not only with the immune system, but also with more generic cellular functions; such defects result in both immunological and non-immunological abnormalities. In addition, there are a number of gain-of-function (GOF) mutations identified such as in PIK3CD. In CARD11 and STAT1 for example, there are both autosomal dominant GOF and autosomal recessive loss of function variants and these different modes of inheritance in the same gene lead to different functional consequences and hence different immunological and clinical phenotypes. The other trend that is increasingly observed is the increase in disorder of immunedysregulation rather than pure immunodeficiency.

The goal of the IUIS Expert Committee on Primary Immunodeficiencies is to increase awareness, facilitate recognition and promote optimal treatment for patients with Primary Immunodeficiencies. In addition to the current report and previous ‘classification table’ publications, the committee has also produced a ‘Phenotypic Approach for IUIS PID classification and Diagnosis: Guidelines for Clinicians at the Bedside,’ which aims to lead physicians to particular groups of PIDs starting from clinical features and combining routine immunological investigations. This will be further updated to include the newly identified defects. Together these contributions will hopefully allow a practical clinical framework for PID diagnosis.

